# How does the alcohol industry attempt to influence marketing regulations? A systematic review

**DOI:** 10.1111/add.13048

**Published:** 2015-08-27

**Authors:** Emily Savell, Gary Fooks, Anna B. Gilmore

**Affiliations:** ^1^ Department for HealthUniversity of BathBathUK; ^2^ UK Centre for Tobacco and Alcohol StudiesBathUK; ^3^ School of Languages and Social ScienceAston UniversityBirminghamUK

**Keywords:** Alcohol industry, corporate policy influence, corporate political activity, framing, marketing policy, marketing regulation, systematic review, tobacco industry

## Abstract

**Aim:**

To systematically review, using a qualitative, narrative synthesis approach, papers examining alcohol industry efforts to influence alcohol marketing policy, and compare with those used by the tobacco industry.

**Methods:**

Literature searches were conducted between April and July 2011, and updated in March 2013. Papers were included if they: made reference to alcohol industry efforts to influence (a) policy debates concerning marketing regulations, (b) new specific marketing policies or (c) broad alcohol policy which included marketing regulations; were written in English; and concerned the period 1990–2013. Alcohol industry political activity was categorized into strategies/tactics and frames/arguments. Data extraction was undertaken by the lead author and 100% of the papers were fully second‐reviewed. Seventeen papers met the review criteria.

**Results:**

Five main political strategies and five main frames were identified. The alcohol industry argues against marketing regulation by emphasizing industry responsibility and the effectiveness of self‐regulation, questioning the effectiveness of statutory regulation and by focusing on individual responsibility. Arguments relating to industry responsibility are often reinforced through corporate social responsibility activities. The industry primarily conveys its arguments through manipulating the evidence base and by promoting ineffective voluntary codes and non‐regulatory initiatives.

**Conclusions:**

The alcohol industry's political activity is more varied than existing models of corporate political activity suggest. The industry's opposition to marketing regulation centres on claims that the industry is responsible and that self regulation is effective. There are considerable commonalities between tobacco and alcohol industry political activity, with differences due potentially to differences in policy contexts and perceived industry legitimacy.

## Introduction

Understanding how large corporations seek to shape health policy has been considerably advanced by the release of millions of internal tobacco industry (TI) documents following litigation in the United States (US) 1, 2, 3. More than 850 peer‐reviewed papers 4 have now been published examining TI political activity, including a growing number of systematic reviews 5, 6, 7, which provide detailed overviews of how the TI seeks to influence policy. Research on alcohol industry (AI) political activity is more case‐study based and, without access to previously confidential industry documents, awareness of the AI's political activity is less developed 8, 9. This is reflected in differences in how public institutions, such as the World Health Organization (WHO), engage with the TI and AI. With regard to the TI, Article 5.3 of the WHO's Framework Convention on Tobacco Control (FCTC, the WHO's first global public health treaty) requires all Parties to protect health policies ‘from commercial and other vested interests of the tobacco industry’ 10 and guidelines for this Article identify the need to actively monitor and expose TI conduct 11. By contrast, the WHO's approach to the AI is more ambiguous; highlighting the importance of protecting the development of health policies from ‘commercial or vested interests’ 12 on one hand, while allowing AI participation 13 on the other. This is despite research illustrating parallels between the political activities of ‘Big Tobacco’ and ‘Big Booze’ 14.

This paper aims to systematically review the tactics (political techniques) and arguments the AI uses to influence public policy relating to alcohol marketing. This focus is chosen for two reasons. First, AI marketing is known to significantly influence drinking initiation and prevalence 15, 16, 17, 18, and restrictions on alcohol marketing are a key element of alcohol control 19, 20, 21. Second, it provides a basis for making comparisons with TI efforts to influence marketing policies which were systematically reviewed in a paper published in 2014 5. The TI review built on existing methods for categorizing corporate political activity in the management literature 22 by classifying both the strategies/tactics and frames/arguments the TI uses in efforts to oppose marketing policies. The present paper develops and applies the TI framework to the AI. It aims to provide a tool for public health advocates and policymakers to understand, predict and potentially counter the AI's efforts to influence alcohol policy. Our approach responds to a recent recommendation that ‘investigations of the strategies of alcohol industry actors may benefit from comparisons with other industries, and particularly with the tobacco industry’ 23.

## Methods

This review aimed to identify all papers (based on either primary or secondary data) that examined AI attempts to influence marketing regulation from 1990 to 2013. The AI comprises large multi‐national companies and tiny specialist brewers, and both on‐ and off‐trade businesses (sales for consumption ‘on’ the premises and ‘off’ the premises, respectively). In this review we include tactics and arguments used by alcohol producers or groups representing producers. Marketing encompasses five key variables: product, promotion, price, place and person 24. Political activity in respect of tax (which affects price) was excluded from the TI systematic review 5 as a systematic review of TI influence on tobacco tax had already been completed 6, so for comparative purposes it was also excluded from this review. However, efforts to influence minimum unit pricing of alcohol were included under price.

The databases Web of Knowledge (which includes Web of Science, BIOSIS Previews and MEDLINE), Business Source Premier and Embase were searched using the string: (corporat* OR industr* OR compan* OR busines* OR firm*) AND (alcohol OR drink) AND (marketing OR advertis* OR sponsor*) AND (regulat* OR policy OR legislat*). The search engine Google was used to identify grey literature and experts were contacted to identify any additional literature (more information is available in the supporting information, Appendix S1). All searches were conducted between April and July 2011, and were updated in March 2013. Searches were limited to papers from 1990 to 2013 and those written in English. The search protocol was developed in conjunction with a qualified librarian.

Initial study inclusion/exclusion criteria were piloted and were discussed extensively between all three authors. The final inclusion/exclusion criteria used in this review can be seen in Box 1. In total, 917 papers were identified, 670 of which were excluded based on their title and abstract alone. 239 papers were downloaded for full analysis (eight papers could not be located despite efforts to contact the authors). 222 papers were excluded for not meeting the inclusion criteria. The remaining 17 papers met all the criteria and were included within the review.

**Box 1 Inclusion and exclusion criteria**

To be included in this review, studies and individual arguments/tactics had to fulfil the following criteria:

- Studies must be written in English.
- Studies must cover the period from 1990 to 2013. In papers that cover both before and after 1990, only those tactics/arguments relating to post‐1990 will be recorded and included within this review. Political activity prior to 1990 is excluded to enable valid comparisons with the findings of our earlier review on the TI 5.
- Studies must look at AI efforts to influence (a) policy debates concerning marketing regulations generally, (b) new specific marketing policies or (c) broader alcohol policy within which marketing is included (information regarding how the industry attempts to circumvent existing regulation will not be included within the review).
- The tactics/arguments covered must be related to one or more of the following: product (for example, packaging, new products/flavours, branding), price* (for example, price promotions, minimum pricing), promotion (advertising including billboards, point‐of‐sale, sponsorship), place (for example, restrictions on advertising near schools) or person (for example, restrictions on advertising or selling to youth).
- Each individual claim made regarding AI tactics/arguments used to influence marketing regulation must be supported directly by verifiable evidence (either a clear citation that could be verified by the authors or a direct quote from an AI official or industry affiliated body).
- Tactics/arguments identified must be implemented directly by the AI or by a group where substantiated evidence suggests that they act on the AI's behalf.
- Tactics/arguments which are noted within the included papers are assumed to have been carried through, in the absence of evidence to the contrary. Tactics/arguments which are shown to only have been planned, and not used, will not be recorded.
- Only tactics/arguments directly related to marketing regulation will be recorded. For example, health warning labels are included as they influence the means of packaging as a marketing tool, but they are excluded if the study only looks at, for example, the wording of the warning, as this does not affect marketing.
- Only tactics/arguments that are clearly detailed in the paper(s) are coded.

*Price in the form of tax has been excluded because tax‐related lobbying was excluded from the systematic review of TI political activity 5, and we aimed to make the TI and AI reviews comparable. Price in terms of price‐based promotions have been included.

Data extraction (supporting information, Appendix S1) was undertaken by the lead author, and 100% of the included papers were second‐reviewed by either the second or third author to check that all the inclusion criteria were met and to agree tactic and argument categorization. Any differences were discussed between all three authors. Disagreements related only to categorization, more often in relation to the categorization of arguments than tactics. Where disagreement occurred, all evidence falling under that particular category was re‐reviewed by all three authors until agreement had been reached. Narrative synthesis was undertaken to combine the evidence from the papers.

Unlike the TI review 5 which was based solely on secondary data, this review is based on both primary and secondary data. Primary data came predominantly from a United Kingdom (UK) parliamentary inquiry into alcohol where four producers and their communications agencies were asked to provide documents relating to five brands and were questioned by Members of Parliament from the Health Select Committee; many additional companies, trade groups and social aspect organizations (SAOs) also provided written evidence 25, 26, 27. Additionally, due to a lack of evidence focusing specifically on AI efforts to influence marketing *regulations*, the review was expanded to include AI influence on marketing *policy debates* alongside their influence on specific marketing regulations, as per the TI review 5.

### Framework of classification

AI political activity was divided into ‘strategies’ containing individual ‘tactics’ (the methods by which a corporation attempts to exert influence) and ‘frames’ containing individual ‘arguments’ (the reasons given by a corporation as to why they oppose one idea or support another). The system of classification developed in an earlier systematic review of TI political activity 5 (which, in turn, had been based partly on Hillman & Hitt's paper 22) was used as an initial framework to code AI political activity. Coding categories (strategies/tactics and frames/arguments) were amended and developed via ‘emergent coding’ 28. This was an iterative process, and the frameworks were only finalized after all of the papers had been reviewed as described above. Once the framework of political activity was finalized, the strategies/tactics and frames/arguments used by the AI were compared to those identified in the systematic review of TI political activity 5.

The geographical distribution of where tactics and arguments were used was also recorded. If the paper included was transnational, the geography of where the individual tactics and arguments were used was listed. For example, the paper by Casswell & Thamarangsi 29 is a transnational study, but the ‘free market economy’ argument was used in France.

## Results

### Geography

In total, 17 papers met our inclusion criteria. A quarter (24%) of the papers focused on Europe, and a further quarter (24%) were transnational (Table 1). No papers focused on AI conduct in South America.

**Table 1:** Geographical location of papers.

*Geographical location*	*Number of papers (%)*	*Papers*
Africa	2 (12%)	Sub‐Saharan Africa 30; South Africa 31
Asia	1 (6%)	Thailand 32
Australasia	3 (18%)	Australia 33, 34, 35
Europe	4 (24%)	UK 27, 36; Netherlands 37; Ireland 38
North America	3 (18%)	USA 28, 39; Canada and USA 40
Transnational	4 (24%)	Transnational 29, 41, 42; Organization for Economic Co‐operation and Development (OECD) 43
Total	17	

### Arguments and tactics

#### AI tactics used to influence marketing regulation

This review identified 20 separate tactics falling under five main strategies (Table 2), which we have termed as follows: ‘information’ (providing or misrepresenting evidence), ‘constituency building’ (forming alliances with other sectors, organizations, or the public to give the impression of larger support for the industry's position), ‘policy substitution, development and implementation’ (proposing, supporting or helping to implement alternative policies), ‘legal’ (using the legal system) and ‘financial incentive or disincentive’ (offering direct or indirect monetary incentives or threatening financial withdrawal) (see further details included in the supporting information, Appendix S1).

**Table 2:** Strategies and tactics used by the alcohol industry when attempting to influence marketing regulation.

*Strategy*	*Tactic*			*Total number of papers, by geography* a
*(total number of uses identified)*				
Information (32)	Direct lobbying (meetings and correspondence with legislators/policymakers)			Africa – 4
				30, 30, 30, 30
				Asia – 1
				32
				Europe – 1
				38
	Indirect lobbying (using third parties, including front groups, to lobby on the industry's behalf)			Africa – 4
				30, 30, 30, 30
	Establishing industry/government collaboration (e.g. via working group, technical group, advisory group)/work alongside policymakers providing technical support/advice/policy development or implementation			Africa – 4
				30, 30, 30, 30
	Evidence	Adding to the evidence base or shaping its understanding	Commissioning, writing (or ghost writing) or disseminating research/publications^b^	Asia – 1
				32
				Europe – 1
				27
				Transnational – 1
				42
			Preparing position papers, technical reports or data on impacts (including economic impact studies)	Asia – 1
				32
				Europe – 1
				38
				Transnational – 1
				42
			Selective citation of industry‐favourable evidence	Europe – 2
				27, 27
				Transnational – 1
				42
			Omission of evidence	Africa – 4
				30, 30, 30, 30
			Removing troubling phrases	Transnational – 1
				42
		Contesting nature of the evidence		Europe – 3
				27, 27, 27
				Transnational – 1
				42
Constituency building (16)	External constituency building		Forming alliances with and mobilising other industry sectors/business/trade organizations	Asia – 1
				32
				N. America – 1
				28
				Transnational – 2
				31, 41
			Media advocacy (press releases, publicity campaigns, public hearings, interviews)	Asia – 1
				32
				Europe – 1
				38
			Forming alliances with or mobilising unions/civil society organizations/ consumers/employees/the public	Asia – 1
				32
				N. America – 1
				28
			Creation of front groups/astroturf/social aspect organizations	Asia – 2
				32, 32
				N. America – 1
				28
	Internal constituency building		Collaboration between companies/development of pan‐industry group or industry trade association^c^	Asia – 1
				32
				Europe – 2
				27, 36
				Transnational – 2
				41, 42
Policy substitution, development and implementation^d^ (28)			Developing/promoting non‐regulatory initiative (generally seen to be ineffective/less effective, e.g. education programmes)	Africa – 4
				30, 30, 30, 30
				Europe – 3
				27, 27, 27
				N. America – 2
				28, 40
				Transnational – 1
				42
			Developing/promoting (new or existing) voluntary code/self‐regulation	Africa – 4
				30, 30, 30, 30
				Asia – 1
				32
				Australasia – 1
				35
				Europe – 6
				27, 27, 27, 36, 37, 38
				N. America – 1
				28
				Transnational – 1
				29
			Developing regulation from scratch and planning implementation	Africa – 4
				30, 30, 30, 30
Legal (3)			Using litigation/raising the prospect of legal action	Asia – 1
				32
				Europe – 1
				29
			Shaping international law	Transnational – 1
				29
Financial incentive or disincentive (1)			Threatening financial withdrawal	Asia – 1
				32

a This column shows the number of times each tactic was used by geography. If a tactic was referred to more than once (in one or more papers) regarding the same policy then it was only counted once; however, if it was referred to more than once about different policies then this was counted separately.

b Including research/publications intended to undermine or misrepresent existing evidence.

c Routine use of a trade association was not counted, industry collaboration must have been more ‘active’.

d Includes efforts to prevent the implementation of anticipated policies

A variety of information strategies were used across multiple jurisdictions. These include direct 30, 32, 38 and indirect 30 lobbying of policymakers and establishing collaborative working arrangements with policymakers 30, and a variety of efforts aimed at shaping and manipulating the evidence base. The latter included commissioning, writing or disseminating research/publications 27, 32, 42 or more technical reports 32, 38, 42, the selective citation 27, 42 and omission of evidence 30, contesting the evidence used to support policy 27, 42 and the efforts to remove ‘troubling’ phrases such as ‘alcohol and other drugs’ from the official lexicon 42. The AI‐funded International Center for Alcohol Policies (ICAP) has played a key part in such efforts: commissioning and publishing a large number of books, monographs, briefing papers, in‐depth reviews of alcohol policy issues, journal papers and policy guides on all manner of alcohol‐related issues 42. These activities have populated the evidence base with non‐peer‐reviewed research which, among other things, tends to highlight the health *benefits* of alcohol 27, 42 and omit evidence of its negative health and social effects 30.

Constituency Building was often linked to indirect lobbying. The AI creates front groups, astroturf organizations 1Astroturf organizations can be defined as ‘fake grassroots organizations usually created and/or sponsored by large corporations to support any arguments or claims in their favor, or to challenge and deny those against them’ 44. or SAOs (such as ICAP 42, the Portman Group 36, The DrinkAware Trust 27 and the Federation on Alcohol Concern of Thailand (FACT) established during the formation of an advertising ban in 2006^32^) to lobby on its behalf 28, 32. It also forms alliances with other industry sectors or trade organizations 28, 32, 41, and civil society organizations, consumers or employees 28, 32 in order to oppose public health measures 41. In Thailand the AI worked with groups such as the Thai Retail Association, the Hotel Association, the Restaurant Association, the Tourism Association and the Marketing Association of Thailand 32, and in the US it reached out to the Federal Trade Commission 28 and built partnerships with government departments, non‐governmental organizations (NGOs), universities, researchers and physicians 28. The AI also uses media advocacy, such as press launches 38 and seminars 32, to shape the news and public agenda.

Policy substitution, used to prevent the implementation of formal marketing regulations, appears to be a key strategy and has been documented globally 27, 28, 29, 30, 32, 35, 36, 37, 38, 40, 42. For example, in Lesotho, Malawi, Uganda and Botswana SAB‐Miller Africa was given *de facto* responsibility for drafting national alcohol policy documents 30. These policy documents focused on self‐regulatory measures, education campaigns, and gave responsibility for the policy's implementation to a National Alcohol Council on which AI representatives served 30. The promotion of self‐regulatory measures is designed to reduce political pressure for and pre‐empt formal regulation and was identified in numerous jurisdictions. For example, we found evidence of voluntary codes being developed and promoted by individual companies 27, 28 and by industry groups in the UK 27, 36, Ireland 38, the Netherlands 37 and transnationally 29. Another technique involves the promotion of non‐regulatory initiatives such as education programmes 27, 28, 30, 40, 42 delivered through stand‐alone websites (for example, SABMiller's www.TalkingAlcohol.com 27) or more (CSR) initiatives. For example, Diageo's Responsible Drinking Fund, which in 2009 claimed to have led or supported more than 130 prevention programmes in more than 40 countries, covering ‘education, public awareness, and responsible retail practices’ 28.

Using or raising the prospect of legal action against a proposed regulation was documented only in Thailand 32 and France 29, but there is also evidence of the AI attempting to shape international trade and investment agreements (specifically the General Agreement on Trade in Services (GATS) with a view to reducing restrictions on alcohol distribution and advertising 29). We also documented one example of the AI using its marketing budget as a lever of policy influence (financial disincentive); in Thailand the AI ‘threatened to withdraw sports sponsorship in retaliation for [an] advertising ban’ 32.

#### AI arguments used to influence marketing regulation

This review identified 20 separate arguments grouped into five main frames (Table 3): ‘regulatory redundancy’ (asserting that proposed policies are unnecessary), ‘legal’ (questioning the legality of policies (the implicit cost for government)), ‘negative unintended consequences’ (direct and indirect compliance costs associated with proposed policies), ‘complex policy area’ (policies, and the issues surrounding them, are presented as highly complicated) and ‘insufficient evidence’ (questioning the strength of evidence supporting policies) (see the supporting information, Appendix S1 for further details).

**Table 3:** Frames and arguments used by the alcohol industry when attempting to influence marketing regulation.

*Frame*			*Argument*	*Total number of papers, by geography* a
*(total number of uses identified)*				
Regulatory redundancy (40)			Industry adheres to own self‐regulation codes/self‐regulation is working well or is better than formal regulation	Africa – 1
				31
				Asia – 1
				32
				Australasia – 3
				33, 34, 35
				Europe – 5
				27, 27, 27, 27, 27
				Transnational – 1
				43
			Industry only markets to those of legal age/is actively opposed to minors using product	Australasia – 1
				34
				Europe – 1
				27
				N. America – 2
				28, 41
			Existing regulation is satisfactory/existing regulation is satisfactory, but requires better enforcement	Asia – 1
				32
				Australasia – 1
				33
				Europe – 3
				27, 27, 38
			Industry is responsible	Australasia – 3
				33, 33, 34
				Europe – 2
				27, 27
				N. America – 1
				41
			Individuals should consume product responsibly/individual‐level approach needed	Africa – 1
				30
				Australasia – 1
				34
				Europe – 2
				27, 27
				N. America – 1
				41
				Transnational – 2
				42, 42
			Industry has positive impact	Africa – 2
				30, 30
				Australasia – 2
				33, 34
				Europe – 1
				27
				N. America – 2
				39, 41
Legal (8)			Infringes legal rights of company (trademarks, intellectual property, constitutionally protected free speech (e.g. US First Amendment), international trade agreements)	Asia – 1
				32
				Europe – 1
				27
				N. America – 1
				41
			Regulation is more extensive than necessary/regulation is disproportionate	Europe – 3
				27, 27, 27
				Transnational – 1
				42
			Interferes with a free market economy	Europe – 1
				27
Negative Unintended Consequences (16)	Economic	Manufacturers	The cost of compliance for manufacturers will be high/the time required for implementation has been underestimated	N. America – 1
				40
			Regulation will result in financial or job losses (among manufacturers)	Asia – 1
				32
			The regulation is discriminatory/regulation will not affect all producers/customers equally	Australasia – 1
				34
				Europe – 3
				27, 27, 27
		Public revenue	Regulation will cause economic/financial problems [for city, state, country or economic area (e.g. European Union)]	Australasia – 1
				34
		Associated industries	Regulation will result in financial or job losses (among retailers and other associated industries, e.g. printing, advertising, leisure)	Asia – 1
				32
				Australasia – 1
				34
	Public health		Regulation will have negative public health consequences	Australasia – 1
				33
				Europe – 2
				27, 27
				N. America – 1
				41
	Other		Regulation could have other negative unintended consequences	Europe – 2
				27, 27
				Transnational – 1
				42
Complex policy area (13)			Complicated/beyond industry's control	Europe – 2
				27, 27
			Collaboration with industry would be beneficial	Africa – 4
				30, 30, 30, 30

				Australasia – 1
				33
				Europe – 2
				27, 38
			Characterizing policymakers and public health actors as authoritarian/denigrating policymakers and public health actors	Asia – 2
				32, 32
				Australasia – 2
				33, 34

Insufficient evidence (8)			There is insufficient evidence that the proposed policy will work/marketing does not cause or change behaviour (it is only used for brand selection and capturing market share), so regulation will have no effect	Asia – 1
				32
				Australasia – 1
				34
				Europe – 3
				27, 27, 38
				N. America – 1
				40
				Transnational – 2
				41, 43

a This column shows the number of times each argument was used by geography. If an argument was referred to more than once (in one or multiple papers) regarding the same policy then it was only counted once; however, if it was referred to more than once about different policies then this was counted separately.

The argument that population‐level health measures are unnecessary (regulatory redundancy frame) is developed through a wide range of mutually reinforcing arguments which rest on industry claims of its own responsibility, its ability to market alcohol responsibly, and its distinction between responsible and irresponsible consumption. This frame included arguments that the AI is responsible 27, 33, 34, 41 (for example that industry always encourages ‘responsible consumption’ 33 and recognizes ‘that responsible drinking is important both to [its] business interests and to society's interests’ 27), that self‐regulatory codes are ‘sufficient’ 31, ‘robust’ 33, ‘effective’ 32, 33, ‘extraordinarily successful’ 35, ‘faster’ 27 and ‘better’ 32, allowing the AI to deal quickly with, and rectify, any complaints or regulatory breeches 27, 34 and close regulatory ‘gaps’ 27, and that the industry markets only to those of legal drinking age 27, 28, 34, 41. Further, the AI appears to overstate the parallels between voluntary and statutory regulation for example by emphasizing the independence of their (industry‐funded) monitoring and adjudication groups 27.

This set of arguments overlaps with claims around personal responsibility and responsible drinking. The AI frequently attempts to shift the blame for alcohol misuse to the consumer and away from their products and marketing 42, arguing that there should be an individual‐level focus on education and the promotion of responsible consumption 27, 30, 42 (and even that AI marketing itself has this aim 41) and that their SAOs such as the DrinkAware Trust and the Portman Group provide information and education so that consumers can make ‘informed judgements’ about how they use alcohol 27; ‘misuse is caused by certain drinkers who clearly misuse alcohol and by some under 18s who are clearly breaking the law. This therefore is not a problem about problem drinks but about problem drinkers’ 27. The focus on a small number of alcohol misusers provides the AI with a frame that has the potential to invalidate the current focus of health policy; the AI argues that population‐level approaches, such as taxation or restrictions on advertising, penalizes moderate drinkers because of a ‘few people’ who consume alcohol in an irresponsible way and that these approaches do not tackle alcohol misuse effectively 27, 34, 42. This supports AI claims that ‘existing regulation is satisfactory’ 27, 33 or that it simply ‘requires better enforcement’ 27, 32, 38; ‘the panoply of powers available to the police and local authorities should be used much more effectively both against individuals who misuse alcohol and those who wilfully seek to break the law in obtaining alcohol underage, as well as against those retailers who sell alcohol irresponsibly’ 27.

The argument that regulations are disproportionate and more extensive than necessary also rests on claims of personal responsibility and the inherent health risks of alcohol. In relation to health warnings in the UK it was stated that it is not ‘necessarily appropriate to have a health warning on a drink of alcohol. Alcohol is not like cigarettes; it is capable of being misused but when drunk in moderation it is perfectly compatible with a healthy lifestyle’ 27. This argument has also been used regarding minimum pricing 27, dealing with alcohol misuse 27 and raising the legal drinking age 42.

Additionally, the AI argues that the industry, its marketing, and alcohol itself, has a positive impact and should therefore not be regulated further: in Lesotho the AI argued that when used in moderation alcohol has a ‘positive role to play in socialisation’ and that the industry is a ‘major contributor to the economy’ 30. In Milwaukee, USA, Miller Brewing argued that a product (beer) which forms a significant part of the local history should not be ‘demonised’ 39. In Australia the AI argued that ‘responsible advertising can have a positive cultural impact’ 33 and highlighted the importance of the AI to communities as ‘funders of events’ 34. And in the UK the wine company Constellation argued that marketing could be used ‘to promote a more responsible approach to alcohol consumption’ 27 (a very similar statement was also made in the US by Philip Morris (tobacco) when they owned Miller Brewing 41).

While actual legal action is rarely reported in the literature (see above), arguments questioning the legality of policies to curb AI marketing are more common. These arguments aim to shift the focus of the debate away from public health and consumer protection, with the AI contesting advertising regulations and minimum pricing proposals under international trade and investment agreements (UK 27 and Thailand 32), national constitutions (USA 41) and international law (UK 27).

The AI often argues that regulation would have negative unintended consequences for manufacturers 27, 32, 34, 40, associated industries 32, 34, the public revenue 34 and public health 27, 33, 41. For example, in both Australia 33 and the UK 27 it was argued that advertising restrictions would make it impossible to introduce new, lower‐strength products to the marketplace, thereby stopping producers from developing and selling healthier products, and in the US the Beer Institute argued that mandated health warnings on alcohol products could ‘undermine the credibility of other government campaigns to provide information about serious risks which are not commonly known’ 41. The AI commonly avoids citing evidence to support such claims, suggesting that the aim may simply be to exaggerate the broader political and economic risks associated with public health measures.

The AI also emphasizes the complications involved in addressing problems associated with alcohol consumption with a view to highlighting the value of industry–government cooperation 27, 30, 33, 38; it claims that it has ‘a unique capacity to access those responsible for promoting and selling alcohol as well as to those who consume their products’ 30 and that ‘partnership working can deliver a more responsible drinking culture’ 27. Although the AI advocate being involved closely in policymaking and alcohol harm reduction initiatives, they also stress that some issues are beyond the AI's control; for example, preventing individual retailers from offering certain price promotions 27 that may be deemed irresponsible. Similarly, the AI regularly characterizes policymakers and public health actors as authoritarian (‘the health lobby's approach is to ban everything, and if it cannot be banned, regulate it severely’ 33) with the Thai government being labelled a ‘dictatorship’ because of an advertising ban 32, and the Australian government being described as a ‘“nanny state” needlessly interfering with people's choices’ 34.

Questioning the strength of evidence favourable to public health policies is another common technique that has been used to oppose advertising bans transnationally 41, in Thailand 32, Australia 34, Ireland 38 and in the UK 27, minimum pricing in the UK 27 and health warning labels in the US 40. This argument is used to reinforce the other arguments made by the AI.

#### Comparison between TI and AI political activity

We identified 13 common tactics used by both the AI and TI 5 when attempting to influence marketing regulation, in addition to five tactics used only by the TI and seven unique to the AI. Similarly we also identified 13 common arguments used by both industries, along with four arguments unique to the TI, and seven (three of which formed the new frame ‘complex policy area’) which had been used only by the AI (see further details included in the supporting information, Appendix S1).

## Discussion

This systematic review illustrates the varied nature of AI political activity used in attempts to influence marketing regulation or marketing‐related policy debates, and highlights similarities with TI political activity.

The results support the findings of the TI review 5 in highlighting the varied nature of industry political activity and provides further confirmation that Hillman & Hitt's 22 model of corporate political activity, which is the most widely cited attempt to analytically categorize the tactics used by corporations, considerably under‐represents the range of tactics corporations use to shape policy outcomes and debates. By identifying tactics/strategies, such as the promotion of self‐regulatory codes and raising the prospect of litigation, the results also challenge Hillman & Hitt's 22 assumption that corporate political activity represents one side of a mutually beneficial exchange relationship in which corporations offer policymakers support and information in return for influencing policy.

The existing literature challenges the validity of many of the arguments identified in this review (see examples in Table 4). For example, despite the AI's assertion that self‐regulatory codes negate the need for formal regulation and that industry collaboration would be beneficial for policymakers, there is no evidence that self‐regulation and industry–government partnerships lead to reductions in alcohol‐related harm 19, 20, 25, 60. Arguing that there is insufficient evidence supporting the need to curb AI marketing and that marketing does not change behaviour is also false as much research has found a significant link between AI marketing and drinking initiation and drinking prevalence 15, 16, 17, 18. Similarly, arguing that the AI does not market to children is misleading, as research shows that AI marketing often targets and appeals to youth and those below legal drinking age 19, 36, 67, 68.

**Table 4:** Veracity of alcohol industry arguments.

*Argument*	*Commentary and examples of evidence*
Industry adheres to own self‐regulation codes/self‐regulation is working well or is better than formal regulation	*Contrary evidence of the former. Strong contrary evidence of the latter* 25, 45, 46, 47, 48, 49, 50, 51, 52, 53
Industry only markets to those of legal age/is actively opposed to minors using product	*Strong contrary evidence of the former. Contrary evidence of the latter* 50, 53, 54, 55, 56, 57
Existing regulation is satisfactory/existing regulation is satisfactory, but requires better enforcement	*Strong contrary evidence*. The available evidence indicates that the contemporary policy environment in Europe and the United States is ineffective in limiting both young people's exposure to alcohol marketing and the general effect of marketing on alcohol‐related harm 17, 47, 54, 56, 58
Industry is responsible	*Strong contrary evidence*. Proxy measures of responsibility such as young people's exposure to alcohol marketing 54 and the weaknesses of industry self‐regulation 25, 47 contradict claims of industry responsibility
Individuals should consume product responsibly/individual‐level approach needed	*Partially supported*. There is some evidence of the effectiveness of individual‐level interventions. Controlled trials of brief alcohol interventions, for example, have reported primarily positive outcomes on weekly drinking and a range of alcohol‐related problems 59. However, this argument is used to imply that population‐based measures are either ineffective or less effective than individual‐level interventions. Studies of the relative effectiveness of different types of policy interventions 17, 60 indicate that there is strong contrary evidence of this contention
Industry has positive impact	*No evidence/not researched*. This assertion rests on narrow claims of social benefits associated with alcohol and the alcohol industry. There are no systematic analyses of the aggregate costs and benefits of current levels of alcohol consumptions (and, by implication, the alcohol industry as presently constituted)
Infringes legal rights of company (trademarks, intellectual property, constitutionally protected free speech (e.g. US First Amendment), international trade agreements)	*No evidence/not researched*
Regulation is more extensive than necessary/regulation is disproportionate	*Strong contrary evidence* 17, 25, 54, 56, 61
Interferes with a free market economy	*Equivocal*. Restrictions on alcohol marketing are designed to manage externalities associated with the alcohol sector
The cost of compliance for manufacturers will be high/the time required for implementation has been underestimated	*No evidence/not researched*. There is no publicly available, independently verified evidence of the compliance costs that accompany marketing regulation
Regulation will result in financial or job losses (among manufacturers)	*No evidence/not researched*. There is no publicly available, independently verified evidence linking alcohol regulation to jobs losses in the industry. In principle, marketing restrictions may negatively affect employment in the alcohol and advertising sectors. Jobs losses that occur as a result of reduced earnings among alcohol producers (resulting from lower consumption) are likely to be offset by the creation of jobs in other parts of the economy which occurs when money which would otherwise be spent on alcohol is disbursed on other products
The regulation is discriminatory/ regulation will not affect all producers/customers equally	*Equivocal*. Marketing regulation need not be discriminatory if properly designed. However, its effects on producers and consumers is not likely to be equally felt
Regulation will cause economic/financial problems (for city, state, country or economic area (e.g. European Union))	*No evidence/not researched*. There is no publicly available, independently verified evidence of these effects
Regulation will result in financial or job losses (among retailers and other associated industries, e.g. printing, advertising, leisure)	*No evidence/not researched*. There is no publicly available, independently verified evidence of the compliance costs that accompany marketing regulation
Regulation will have negative public health consequences	*No evidence/not researched*. There is no evidence to suggest that alcohol restrictions will have aggregate negative public health consequences
Regulation could have other negative unintended consequences	*No evidence/not researched*. Risks of negative unintended consequences resulting from policy innovation cannot be discounted. The important policy questions, however, concern the probability of these risks and whether negative outcomes associated with policy innovation outweigh its social benefits. There is no publicly available, independently verified evidence on these issues
Complicated/beyond industry's control	*Contrary evidence*. Alcohol‐related harm is multiple‐causal. However, when viewed against studies on the relationship between marketing and consumption (see above) studies outlining the volume 46 and focus of industry marketing 56, 62 suggest that marketing is a key driver of aggregate levels of consumption and, therefore, alcohol‐related harm
Collaboration with industry would be beneficial	*Contrary evidence* 47, 63, 64
Characterizing policymakers and public health actors as authoritarian/denigrating policymakers and public health actor	*Unable to comment*
There is insufficient evidence that the proposed policy will work/marketing does not cause or change behaviour (it is only used for brand selection and capturing market share), so regulation will have no effect	*Strong contrary evidence* 16, 17, 65, 66

This review identifies marked similarities between TI and AI political activity 5. Differences in the observed political activities of each industry may be due to a number of factors. First, raising concerns about compensation or debating which body has power to regulate (as the TI has done), for example, are arguments likely to be made in the face of impending regulation by companies which have lost the ability to exercise insider influence over policy discussions, reflecting greater TI regulation and differences in alcohol and tobacco policy contexts. Second, differences in framing may reflect variations in how different industries make similar points. For example, while the AI may not directly contest the health impacts of alcohol consumption, questions about the degree of harm caused by alcohol consumption are implicit in claims concerning individual responsibility and the health *benefits* of alcohol consumption. Third, differences may reflect differences in access to data; because of the availability of TI documents, information on lower visibility political activity, such as raising the prospect of legal action, is more available on the TI. Finally, differences may reflect the broader inclusion criteria used for the AI review (i.e. covering policy debates concerning marketing regulations generally rather than just new specific marketing policies), the inclusion of both primary and secondary evidence in the AI review, and differences in alcohol and tobacco policy contexts. For example, despite not being identified in the TI review there is evidence of the TI attempting to shape the evidence base 69, 70, 71, 72, influencing international regulations 73, 74, and focusing on individual responsibility 75.

Consistent with the TI review findings 5, many of the individual arguments fall within a larger ‘cost–benefit’ meta‐frame which promotes the economic and social costs of proposed public health policies and underplays their benefits. Arguments claiming that regulation is more extensive than necessary and likely to produce negative unintended consequences are used to increase uncertainty about the likely benefits of regulation, and highlight the potential future costs for the industry, retailers, and the public revenue. This is also observed through the omission of a ‘health’ frame 76; this review found little evidence of the AI making reference to the dangers of drinking alcohol (only in terms of references to ‘problem drinkers’), although multiple examples of the AI highlighting the potential health *benefits* of alcohol consumption were identified 27, 42. The review also found that many arguments were supported by CSR activities. CSR tends to be used strategically by an industry to prevent the introduction of legislation 77. By acting as vehicles for the promotion of arguments, CSR activities such as self‐regulatory codes work politically as agenda‐setting devices which frame issues and shape policy debates 77. The AI's emphasis on CSR highlights its value in maintaining industry credibility and forming relationships (CSR partnerships are likely to create further opportunities for cooperation 78) ahead of regulation.

### Strengths and limitations

This review has a number of limitations. First, although a broad search strategy and search string was used when initially identifying papers it is still possible that some relevant papers may have been missed. To minimize this, we worked with a librarian, searched online research repositories, and contacted experts in the field to identify additional papers. Second, interpretive coding of arguments and tactics is ultimately subjective. To mitigate this, all three authors reviewed and re‐reviewed the coding at various points during the systematic review process and second‐reviewed all the included papers to ensure consistency. Third, the identification of tactics and arguments, and the jurisdictions in which they are used, is dependent upon the available literature, its quality, and any publication bias. As such, it is possible that some tactics and arguments are not identified in or used more frequently than the literature would suggest. Closely related to this is the fact that the review focuses only on marketing policy, and the AI may use a more diverse set of tactics and arguments in other policy areas. For these reasons the number of papers listed next to each tactic and argument (the ‘count’) should be used only as an indication of the reliance the AI places on particular tactics and arguments. Finally, due to limited information in the papers identified, it was not possible to reliably determine which tactics or arguments were most persuasive or successful in defeating marketing‐related regulations.

The main strength of this review is its systematic approach, which provides a comprehensive and geographically diverse overview of AI tactics and arguments. Its attempt to rigorously categorize industry strategies/tactics and frames/arguments is, to our knowledge, along with our first paper 5, the first attempt to do so. While care needs to be taken in assuming that tactics and arguments used in one jurisdiction will be used elsewhere, this and our previous review 5 suggest that the findings will be broadly applicable across different jurisdictions.

### Implications for policy, practice and research

This systematic review has identified strategies/tactics and frames/arguments used by the AI between 1990 and 2013 to shape policy debates and prevent the implementation of restrictions on alcohol marketing. Policymakers need to be aware of these in order to understand how the AI may try to influence the policymaking process, and public health actors can use this information to prepare effective counter strategies and arguments. This review has also confirmed substantial commonalities between AI and TI political activity: particularly the use of obfuscating tactics such as misrepresenting the evidence base and using third parties and front groups to lobby. The similarities suggest that alcohol policy may benefit from reproducing efforts in tobacco control aimed at excluding corporate actors from the policy process and enhancing transparency. Additionally, as differences between the two industries are likely to be due, at least in part, to differences in alcohol and tobacco policy contexts, the findings from the TI review 5 may provide an indication of how AI political activity is likely to develop under conditions of increased regulatory risks.

The current review has further developed the frameworks for classifying corporate political activity outlined in the earlier TI review 5, and shown the policy and scholarly value of applying them to other industries. Future work could apply these frameworks to other industries or policy areas. Based on limitations in the studies reviewed, we again recommend that future research on corporate policy influence should, where possible, include contextual information, ensure all claims made within the paper are supported by empirical evidence, and that the receptivity of stakeholders to and the success or failure of individual tactics and arguments are recorded.

### Declaration of interests

None.

## Supporting information




**Table S1** Description of Strategies and Frames
**Table S2** Use of tactics by the tobacco industry (TI) and alcohol industry (AI)
**Table S3** Use of arguments by the tobacco industry (TI) and alcohol industry (AI)
**Table S4** Summary of studies relating to alcohol industry (AI) attempts to influence marketing‐related regulation.
**Table S5** Searches completed and the number of articles returned

Supporting info item
