# Supplementary material for: How does the alcohol industry attempt to influence marketing regulations? A systematic review
Source: Addiction. 2015 Aug 27;111(1):18–32. doi: 10.1111/add.13048 (PMC4681589; doi:10.1111/add.13048)
Supplement: Supplementary file 1 — Supporting info item [file ADD-111-18-s001.pdf]

Online Appendix

Taxonomies 2
Table 1: Description of Strategies and Frames 2
Table 2: Use of tactics by the tobacco industry (TI) and alcohol industry (AI) 5
Table 3: Use of arguments by the tobacco industry (TI) and alcohol industry (AI) 8
Data Extraction 10
Table 4: Summary of studies relating to alcohol industry (AI) attempts to influence marketing-related regulation. 10
Explanation of systematic searches 129
Table 5: Searches completed and the number of articles returned 129

Taxonomies

Table 1: Description of Strategies and Frames

Category	Description
**Strategies**	
**Information**	Policymakers face considerable capacity problems in collating information relevant to the economic and health consequences of proposed policies. Businesses enjoy information advantages relative to policymakers partly because of their capacity to gather information (on, for example, employment figures or product ingredients and their impacts) first-hand through research or direct observation[1]. Businesses take advantage of these information asymmetries by supplying policymakers with information about the likelihood of adverse effects of proposed policy measures. Exchange theory assumes that information supplied by business represents a form of subsidy, which invariably aids optimal policy outcomes[2]. In practice, however, policymakers often lack the information necessary to assess the accuracy or veracity of industry predictions[1]. This, and commercial pressures to shape policy to their advantage, provide companies with incentives to overstate their case and misrepresent policy relevant information. As the tactics summarised in this systematic review illustrate, the AI exploits this information inequity by providing policymakers with false or misleading information by understating the health benefits of a proposed policy and overstating its social and economic consequences.
**Constituency building**	Constituency Building involves generating support of other social actors (other businesses, trade associations, organisations, or individuals) in order to give the impression of a large support-base for a company or industry's policy preferences[3]. Because the outcome of policy discussions in contemporary democracies is often determined by the success or failure of efforts to enlarge their scope, constituency building is a key political strategy of both public health and business actors. Moreover, constituency building can also reinforce other political practices, such as lobbying. Both elected and non-elected officials attach a premium to constituent support. Business approaches constituency building in three key ways: forming alliances with other organisations (such as trade associations, unions, or organisations representing other industry sectors); mobilising the public (through publicity campaigns, increasingly through digital media, and press releases); and via

	false alliances built through the creation of front groups and astroturf organisations[4].
**Financial incentive or disincentive**	Businesses seek to influence policymaking through a range of financial inducements and disincentives. Inducements range from campaign finance and other political donations, to more 'under-the-counter' practices, such as offers of employment to policymakers, gifts, and the provision of entertainment[4].<br><br>Financial disincentives recognise the structural dependence of governments on business and policymakers' assessment of their electoral success on good economic performance. Such disincentives can involve capital flight (companies moving operations overseas or, on a more modest scale, withdrawing from, for example, sponsorship contracts) and capital strike (where companies refuse to invest).
**Policy substitution, development and implementation**	In addition to financial inducements, offers of support (Constituency Building), and information subsidies, business actors can also provide policy subsidies, where alternative policies are developed as a substitute for proposed policies. Policy substitution is attractive to policymakers as it reduces the administrative costs associated with developing and implementing policies, whilst still appearing to meet their policy objectives. Voluntary codes represent the most obvious policy subsidy, although companies can also offer to develop alternative statutory (or other more formal) policies where policymaking capacity is weak. The risk of such policy subsidies for policymakers is that the alternatives suggested are less effective at promoting public health.
**Legal**	Business is likely to use or raise the prospect of legal action when proposed policies are imminent and other, less adversarial approaches have failed[5, p.23]. Litigation (and the prospect of litigation) work by raising the (perceived and actual) costs associated with implementing regulation, and underline the immediate fiscal advantages of voluntary codes and regulatory policies acceptable to the industry.<br><br>International trade and investment agreements[6] also represent an important means of challenging health measures. Shaping such agreements represent a strategically important way of remotely shaping health policy[6].

Frames	
**Negative Unintended Consequences**	The industry uses a wide range of arguments to highlight the potential negative effects of regulation. These 'negative unintended consequences' include economic impacts (such as financial or job losses for manufacturers, losses to the public revenue, and losses to associated industries such as the hospitality and advertising industries), public health impacts (where the risk of regulation causing harm to health through, for example, increased consumption, is highlighted), and a wide range of other impacts, including setting a precedent for other types of products and causing confusion amongst customers. The argument that proposed regulations will increase illicit trade feeds into many of the other 'negative consequence' arguments, as increases in illicit trade can have both economic and public health impacts (by making the product cheaper and more available)[4].
**Legal**	Legal arguments highlight the illegality of proposed policies. They generally aim to shift the focus of the debate away from public health and consumer protection, and instead highlight the potential administrative costs of new policies.
**Regulatory Redundancy**	The industry uses a number of arguments which seek to emphasise that proposed policies are unnecessary, and liable to waste public funds. These points are conveyed through, for example, industry claims that it is capable of self-regulation, that existing regulation is satisfactory (and simply in need of better enforcement), and that industry is responsible and doesn't need to be formally regulated.
**Insufficient Evidence**	The industry uses a number of arguments aimed at questioning the strength of the evidence base behind proposed health policies. Such arguments aim to increase scepticism of the likely benefits of proposed policies and support arguments from other frames, which emphasise the costs and underplay the benefits of public health policies.
**Complex Policy Area**	Complexity is a recurring theme in business lobbying. The concept is primarily used to cast doubt on academic research and highlight the difficulties involved in addressing multi-causal social problems with relatively focused policy interventions. In practice, these claims are designed to build a case for the relative effectiveness of voluntary codes, which, in contrast to the blunt tool of statutory intervention, is sufficiently flexible to reflect the complexity of the evidence relating to alcohol related harm.

Table 2: Use of tactics by the tobacco industry (TI) and alcohol industry (AI)

Strategy	Tactic			Evidence of use	
				TI	AI
Information	Direct lobbying (meetings and correspondence with legislators/policymakers)			√	√
	Indirect lobbying (using third parties, including front groups, to lobby on the industry's behalf)			√	√
	Establishing industry/government collaboration (e.g. via working group, technical group, advisory group) / work alongside policymakers providing technical support/advice / policy development or implementation			√	√
	Evidence	Adding to the evidence base or shaping its understanding	Commissioning, writing (or ghost writing), or disseminating research/publications	√	√
			Preparing position papers, technical reports or data on impacts (including economic impact studies)	√	√
			Selective citation of industry-favourable evidence		√
			Omission of evidence		√
			Removing troubling phrases		√
		Contesting nature of the evidence			√
Constituency building	External constituency building		Forming alliances with and mobilising other industry sectors/business/trade organisations	√	√
			Media advocacy (press releases, publicity campaigns,	√	√

		public hearings, interviews)		
		Forming alliances with or mobilising unions/civil society organizations/ consumers/employees/the public	√	√
		Creation of front groups/astroturf/social aspect organisations	√	√
	Internal constituency building	Collaboration between companies / development of pan-industry group or industry trade association	√	√
Policy substitution, development and implementation		Developing/promoting (new or existing) voluntary code / self-regulation	√	√
		Developing/promoting non-regulatory initiative (generally seen to be ineffective/less effective, e.g. education programmes)	√	√
		Developing/promoting alternative regulatory policy	√	
		Developing regulation from scratch and planning implementation		√
Legal		Using litigation / raising the prospect of legal action	√	√
		Pre-emption	√	
		Shaping international law		√
Constituency fragmentation and destabilization		Preventing the emergence of, neutralising and/or discrediting potential opponents (individuals, organisations or	√	

	coalitions)		
Financial incentive or disincentive	Providing current or offering future employment to those in influential role	√	
	Gifts, entertainment or other direct financial inducement	√	
	Threatening financial withdrawal		√

Table 3: Use of arguments by the tobacco industry (TI) and alcohol industry (AI)

Frame	Argument	Evidence of use	
		TI	AI
Regulatory Redundancy	Industry adheres to own self-regulation codes / self-regulation is working well or is better than formal regulation	√	√
	Industry only markets to those of legal age / is actively opposed to minors using product	√	√
	Existing regulation is satisfactory/Existing regulation is satisfactory, but requires better enforcement	√	√
	Industry is responsible		√[1]
	Individuals should consume product responsibly / individual-level approach needed		√
	Industry has positive impact		√
Legal	Infringes legal rights of company (trademarks, intellectual property, constitutionally protected free speech (e.g. US First Amendment), international trade agreements)	√	√
	Regulation is more extensive than necessary / regulation is disproportionate	√	√
	Body doesn't have the power to regulate / it's beyond their jurisdiction	√	
	Regulation will cause an increase in compensation claims	√	
	Interferes with a free market economy		√

[1] Although the 'industry is responsible' argument was not identified in the TI review, similar arguments such as adhering to self-regulation and not marketing to minors were identified

Negative Unintended Consequences	Economic	Manufacturers	The cost of compliance for manufacturers will be high / the time required for implementation has been underestimated	√	√
			Regulation will result in financial or job losses (among manufacturers)	√	√
			The regulation is discriminatory / regulation will not affect all producers/customers equally	√	√
		Public Revenue	Regulation will cause economic/financial problems (for city, state, country or economic area (e.g. European Union))	√	√
		Associated industries	Regulation will result in financial or job losses (among retailers and other associated industries, e.g. printing, advertising, leisure)	√	√
	Public Health		Regulation will have negative public health consequences	√	√
	Illicit Trade		Regulation will cause an increase in illicit trade	√	
	Other		Regulation could have other negative unintended consequences	√	√
Complex Policy Area			Complicated/beyond Industry's control		√
			Collaboration with Industry would be beneficial		√
			Characterising policymakers and public health actors as authoritarian/denigrating policymakers and public health actors		√
Insufficient Evidence			There's insufficient evidence that the proposed policy will work / marketing doesn't cause or change behaviour (it's only used for brand selection and capturing market share), so regulation will have no effect	√	√
			The health impacts of consumption remain unproven	√	

Data Extraction

Table 4: Summary of studies relating to alcohol industry (AI) attempts to influence marketing-related regulation.

Study	Geography	What policy is the AI attempting to influence?	Tactics used	Arguments used
B&T Magazine, 2012 [7]	Australia	General marketing –<br><br>A stoush has broken out between the newly-formed Alcohol Advertising Review Board (AARB) and the AANA, with the body representing some of the biggest advertisers in the country claiming that the AARB is attempting to introduce greater alcohol advertising regulation by stealth.		Jeremy Griffith (Corporate affairs director, Carlton United Brewers):<br><br>As tempting as it is to allow the health lobby to decide what is funny and proper, we think the current approach to advertising self-regulation gets it right. The Alcoholic Beverages Advertising Code (ABAC) is robust and effective and meets with community standards. The health lobby's approach is to ban everything, and if it cannot be banned, regulate it severely. It pays no regard to whether the current system actually works. The ABAC is

Study	Geography	What policy is the AI attempting to influence?	Tactics used	Arguments used
				well enshrined in the industry – from the creative and scripting stages to the filming and placement process. It achieves what it intends – to ensure that alcohol is marketed responsibly. As a result, only 4% of complaints received by the ASB each year relate to alcohol advertising. Most importantly, the system has teeth. As signatories to the code, when complaints to the ABAC are upheld, we pull those campaigns. This can cost millions of dollars. We always encourage responsible consumption of our beers, but we certainly won't step away from using humour or nice-looking amber liquid in carefully frosted glasses to sell it. Finally, we welcome anybody to visit us to review our ads and approval process to

Study	Geography	What policy is the AI attempting to influence?	Tactics used	Arguments used
				ensure we are completely aligned with community standards.<br><br>Alina Bain (Director of codes, policy and regulatory affairs, Australian Association of National Advertisers):<br><br>There is no need for greater regulation of alcohol advertising as there are already a number of regulatory protections in place in terms of messaging and placement of alcohol advertising. The current self-regulatory system is effective and underpinned by a responsive and transparent complaints handling system. A system that delivers responses to consumer complainants within 30 days and covers all forms of advertising, including new media. The Alcoholic Beverages Advertising Code

Study	Geography	What policy is the AI attempting to influence?	Tactics used	Arguments used
				(ABAC) is co-regulatory with a government representative on the management committee. ABAC'S independent adjudication panel is led by former Attorney General Professor the Hon Michael Lavarch, and is run at arm's length from the industry. It should be noted that alcohol advertisements have to be approved against the code before they go to market, by an independent pre-vetter. The low level of consumer complaints about alcohol advertising demonstrates the system is delivering for the public. According to the Advertising Standards Bureau, alcohol advertising accounts for just 3.78% of all complaints received.<br><br>Leela Sutton (External relations director, Lion):

Study	Geography	What policy is the AI attempting to influence?	Tactics used	Arguments used
				A couple of months ago, mid-strength beer XXXX GOLD became Australia's leading beer. Most new beers released by the major brewers are now brewed at considerably less than 5% alcohol and over the last two decades there has been a significant reduction in average alcohol content. Without marketing and sports sponsorship it would be close to impossible to establish these new beers in the marketplace. While irresponsible advertising should not be tolerated, responsible advertising can have a positive cultural impact. The inflexible ideology of those behind the AARB would have us believe that sports sponsorship will make Australians think they will achieve sporting success if they drink - but we don't

Study	Geography	What policy is the AI attempting to influence?	Tactics used	Arguments used
				think they are that dim. We associate our brands with sport because the audience is mainly adult males and they tend to enjoy a beer when they watch it. Alcohol marketing is already highly regulated. Advertisements have to be approved by an independent pre-vetter before they go to market and, since 2008, the ABAC complaints panel has made 151 determinations with 35% upheld. All forms of regulation need to move with the times and the ABAC system must continue to evolve with the changing media landscape. Rather than confusing genuine complainants and wasting money on PR stunts, those behind the AARB would be better served working with industry to reduce alcohol misuse through practical

Study	Geography	What policy is the AI attempting to influence?	Tactics used	Arguments used
				solutions the community is likely to support.
Bakke and Endal, 2010 [8]	Sub-Saharan Africa	National alcohol policy drafts (not just marketing)	A comparison was conducted of four draft National Alcohol Policy documents from Lesotho, Malawi, Uganda and Botswana. The comparison indicated that the four drafts are almost identical in wording and structure and that they are likely to originate from the same source.<br><br>The properties information of two of the MSWord documents indicated that the 'author' was 'mramsay' from the 'company' SABMiller Africa Asia, possibly Mitch Ramsay, Policy and Issues Manager, SAB-Miller Africa. His later comment that 'at the invitation of the workshop attendees, I was invited to prepare a record of the workshop policy proposals in local policy format' may explain this (RamsayM., unpublished observations).<br><br>The initiative to develop an alcohol policy came from the Ugandan parliament and the task was entrusted to the Ministry of Health. The alcohol industry came on board only later [12]. In the other cases the initiative seems to have come from the drinks	...the Lesotho draft policy begins with the premise that the National Alcohol Policy 'recognises the role alcohol plays in Lesotho, both in terms of its social and economic contribution and in terms of its significant capacity, when misused, to impose unacceptable costs on individuals and the community as a whole'. This and other documents emphasize specifically that: '[t]he Government acknowledges that alcohol enjoys popularity and a place of significance in Lesotho society. Alcohol when used in moderation has a positive role to play in socialisation and the industry is a major contributor to the economy of Lesotho'. One of their key guiding principles

Study	Geography	What policy is the AI attempting to influence?	Tactics used	Arguments used
			industry.<br><br>All four documents have the same core of policy measures and some key formulations that we would expect the alcohol industry wants to see included. The Lesotho and Malawi documents are almost identical. The Botswana document is also very similar to the two discussed above. Some sections were moved, some removed and other changes had been incorporated. The Uganda document differed somewhat more from the three others. Many of the paragraphs are the same, although the structure is somewhat different.<br><br>The documents describe that senior representatives of government agencies, NGOs and representatives of civil society groups have been invited to attend workshops and consultations. Reportedly, the workshops were facilitated by Mitch Ramsay of SABMiller and Mr Keith Evans. In another capacity Mr Evans is the Director, Primary Health Care and Drug Strategy, South Australian Department of Health. He is also listed as a consultant to the Washington-based International Center for	enshrines 'the right of the alcohol industry to conduct legitimate and legal business in a responsible way'.<br><br>The last part of the draft guidelines for implementation places the responsibility for implementation of the policy with a 'National Alcohol Council', on which representation is reserved for the industry: '[The Council] will draw its membership from Government Officials, representatives of the academic and Public Health Community, representatives of the Non-Government Sector and Civil Society and representatives from the Beverage Alcohol Industry'. According to the proposal, the council will also be responsible for monitoring and for reviewing the National Alcohol Policy every 4–5 years. The policy drafts state the reason

Study	Geography	What policy is the AI attempting to influence?	Tactics used	Arguments used
			Alcohol Policies (ICAP). ICAP is funded by the largest multi-national beverage alcohol producers ... to operate as an agent for industry interests in global and national policy arenas. Neither the draft policy documents nor the other sources analysed indicate clearly whom Mr Evans represents.<br><br>According to Mr Evans himself, he acted as an independent facilitator in the workshops at the request of the relevant government departments of the countries concerned.<br><br>In personal communication and later written comments (unpublished), Mr Mitch Ramsay, Policy and Issues Manager of SABMiller, informed that SABMiller made it possible for Keith Evans to facilitate the workshops.<br><br>The last part of the draft guidelines for implementation places the responsibility for implementation of the policy with a 'National Alcohol Council', on which representation is reserved for the industry: '[The Council] will draw its membership from Government Officials, representatives of the academic and Public Health Community, representatives of the Non-Government	for the industry's integral involvement in the policy process by citing its 'vested interest' in reducing alcohol misuse: 'The Government will encourage active participation by all levels of the beverage alcohol industry as a key partner in the policy formulation and implementation process. The beverage alcohol industry has a vested interest in ensuring that alcohol misuse is substantially reduced, and has a unique capacity to access those responsible for promoting and selling alcohol as well as to those who consume their products'.

Study	Geography	What policy is the AI attempting to influence?	Tactics used	Arguments used
			Sector and Civil Society and representatives from the Beverage Alcohol Industry’. According to the proposal, the council will also be responsible for monitoring and for reviewing the National Alcohol Policy every 4–5 years. The policy drafts state the reason for the industry’s integral involvement in the policy process by citing its ‘vested interest’ in reducing alcohol misuse: ‘The Government will encourage active participation by all levels of the beverage alcohol industry as a key partner in the policy formulation and implementation process. The beverage alcohol industry has a vested interest in ensuring that alcohol misuse is substantially reduced, and has a unique capacity to access those responsible for promoting and selling alcohol as well as to those who consume their products’.<br><br>According to the facilitators a tripartite model addressing supply, demand and harm reduction strategies was presented (Evans K., unpublished observations) and there were ‘significant discussions about population-based measures and references to relevant WHO sponsored research’ (RamsayM., unpublished observations).What-ever the	

Study	Geography	What policy is the AI attempting to influence?	Tactics used	Arguments used
			content of these discussions in the workshops, the draft national alcohol policies take essentially the same approach proposed in ICAP's publication *Drinking in Context*, where the emphasis rests upon the need to manage drinking patterns and strengthen industry/government/public health partnerships... The draft policy documents are devoid of any reference to *Alcohol: No Ordinary Commodity* or other compilations of the international evidence base on alcohol prevention developed by independent alcohol researchers working on behalf of the WHO.<br><br>Priority area four—patterns and availability—basically upholds the need to 'develop and implement a transparent self-regulatory system by the alcohol beverage industry' and conduct public education campaigns.<br><br>In this section there is also a mention of the need to regulate alcohol promotions concluding by subscribing to the preferred industry approach: 'The Government supports the need for self-regulation by the alcohol beverage industry as the most	

Study	Geography	What policy is the AI attempting to influence?	Tactics used	Arguments used
			suitable way to manage marketing and promotions'<br><br>... according to Mr Ramsay (unpublished observations), social and economic deprivation were reportedly discussed in the workshops, those perspectives are not addressed in the policy documents, nor are other specific challenges to developing countries such as alcohol and its relations to key issues such as human immunodeficiency virus/acquired immune deficiency syndrome (HIV/AIDS), gender-based violence, child rights and others<br><br>We have documented above how the focus of the industry's draft policy is towards the economic and social contribution of alcohol in the society. The key issues and policy measures in the documents indicate that they promote industry self-regulation, for instance with regard to marketing. Their fear of restrictions on alcohol advertising and other marketing activities at a time when the multi-national beer producers are increasing their presence in many African countries might be an important impetus for the present industry initiatives. As long as they	

Study	Geography	What policy is the AI attempting to influence?	Tactics used	Arguments used
			can relegate marketing activities to self-regulation, they can use advanced marketing techniques to promote drinking among new segments of the population in these countries.	
Bond et al, 2009 [9]	Transantional	General marketing restrictions: advertising and labelling regulations	Partnerships and consultation between alcohol, tobacco and allied companies have also assisted in the development of industry strategies to resist legislative and regulatory measures. To counter advertising and labelling regulations, the MBC five year plan (1992-1996) intended to "coordinate response efforts to proposed restrictions with the entire alcohol beverage industry.	As part of PM's defence of advertising, a recurring theme is reference to the US First Amendment for protection against advertising restrictions on both alcohol and tobacco products. "Truthful advertising about legal products is protected by the First Amendment". PM's position on advertising restrictions on alcohol is:<br><br>"What about proposals to restrict advertising? Numerous credible studies conclude that advertising does not cause alcohol abuse; therefore ad bans will not stop it. Studies conducted by government and independent researchers conclude advertising affects

Study	Geography	What policy is the AI attempting to influence?	Tactics used	Arguments used
				brand performance, not consumption or abuse”<br><br>The BI claim that alcohol advertising “does not cause alcohol abuse or underage drinking. Contrary to what some believe, there is no evidence to support that link” . The Distilled Spirits Council of the United States (DISCUS) also maintains that “there is no direct linkage between exposure to alcohol ads and the total consumption of alcohol beverages” . DISCUS further states: “The economic effects of a restrictive ban on alcohol advertising would be discriminatory and would adversely affect the legitimate right of an industry, which maintains a good record of social responsibility, to market its products”. PM argue that the company advertises its beer to encourage consumers of legal drinking age to select

Study	Geography	What policy is the AI attempting to influence?	Tactics used	Arguments used
				the Miller brand over other brands. It also aims to "remind drinkers about the importance of consuming products responsibly"<br><br>A further alcohol industry concern identified from the documents related to mandated labelling of alcohol products including health warnings. An 'Advertising Warning Legislation' information sheet developed by the BI asks readers to "consider the facts". The BI asserts:<br><br>"There is no empirical evidence that warnings would have any effect on the drinking patterns of Americans of any age, especially among alcohol abusers. Worse, these types of warnings could undermine the credibility of other government campaigns to provide information about serious risks

Study	Geography	What policy is the AI attempting to influence?	Tactics used	Arguments used
				which are not commonly known."<br><br>PM's argument on mandated health warnings is:<br><br>"Could you detail your opposition to legislation which would mandate health warnings on alcohol advertising? Numerous studies demonstrated that these warning messages do not reduce alcohol abuse among any segment of the population. These proposals divert attention and resources from other effective programs that we support to combat abuse. Advertising does not cause alcohol abuse, and there is no evidence to support that link"<br><br>In response to concerns about under-age consumption, the 2000 CEO Issues Book emphasised that MBC advertise and market their

Study	Geography	What policy is the AI attempting to influence?	Tactics used	Arguments used
				products to appeal only to adults of legal drinking age. They also maintained that advertising products to underage people has no effect:<br><br>"Doesn't Miller's advertising appeal to underage people? While we understand your scepticism, sound research shows that advertising has a negligible effect on youth consumption; on its own, advertising doesn't make young people drink"
Casswell and Thamarangsi, 2009 [10]	Transnational	General marketing	Alcohol policy has been safeguarded from the effect of trade agreements in other situations. France's alcohol policy law, Loi Evin, which restricts alcohol advertising, was challenged by the European Commission and the UK. However, the European Court decided that the law was justified on the grounds that it protected health and was an effective strategy. Similarly, after a challenge to Sweden's regulations on alcohol advertising, Sweden was able to retain most of its restrictions by rewriting the legislation	

Study	Geography	What policy is the AI attempting to influence?	Tactics used	Arguments used
			to state clearly that the policy was necessary to achieve public health goals.<br><br>Alcohol producers are well organised and effective lobbyists for industry-friendly policies both internationally and nationally. Representatives of the global alcohol industry, especially the distilled spirits sector, were strong supporters of trade treaties that expanded their access to rapidly emerging markets. The World Spirits Alliance lobbied for the General Agreement on Trade in Services (GATS), seeking liberalisation or elimination of barriers to tariffs and non-tariffs, including all restrictions on distribution and advertising.<br><br>A major focus of industry lobbying is to campaign against effective strategies and for ineffective strategies— examples in which this lobbying has been successful are documented. For example, in Brazil, at a time when the government was reviewing the law on advertisement of alcohol products, the largest brewer (AmBev) initiated a publicity campaign against driving while under the influence of alcohol, a partnership with taxi drivers, and an educational programme	

Study	Geography	What policy is the AI attempting to influence?	Tactics used	Arguments used
			against drinking by minors—none of which has evidence for effectiveness.<br><br>ICAP was established in 1995, and has promoted industry interests in a relative absence of international public health activity. ICAP's activities are similar to those of organisations representing other globalised industries—including participation in scientific and policy agendas; and support for research, publications, and conferences. A major ICAP focus is on the development of working partnerships with alcohol research and public health. ICAP's position is that the industry has a part to play in developing alcohol policies in emerging markets. In 2006 and 2007, three regional meetings promoting voluntary codes in advertising were held in key emerging alcohol markets—Asia Pacific (China, Vietnam, Laos, India, and Thailand), Africa, and Latin America. Promotion of voluntary codes and strong arguments against regulation of marketing has been a major focus for ICAP and for the industry in general. A clear distinction can be made between the policies promoted in ICAP publications and those that are assessed as	

Study	Geography	What policy is the AI attempting to influence?	Tactics used	Arguments used
			effective in non-industry funded reviews.	
Fogarty and Chapman, 2012 [11]	Australia	General marketing		Advertising restrictions: unnecessary for a responsible industry<br><br>In this news frame, advertising restrictions were positioned as unwarranted by a responsible drinks industry that was said to be already actively managing alcohol risk. Such framing emphasised existing guidelines as more than adequate, raised examples of the industry reacting swiftly to complaints and policing its own promotional material, denied that the industry caused harm directly or targeted children and stressed their importance to community as funders of events. This angle sought to re-frame the public health position on advertising

Study	Geography	What policy is the AI attempting to influence?	Tactics used	Arguments used
				restrictions as unnecessary punishment of moderate drinkers for the behaviour of a few people and cheap political point-scoring at the industry's expense.<br><br>Advertising restrictions as an attack on legitimate commercial activity<br><br>This frame suggested that introducing greater regulation of alcohol advertising would be an attack on the advertising industry. Negative consequences such as job losses, erosion of commercial freedom, the stifling of creativity and negative impact on the economy were highlighted. Such framing included calls to lobby the government directly to oppose the policy. No mention was made of alcohol-associated harms

Study	Geography	What policy is the AI attempting to influence?	Tactics used	Arguments used
				and supporters of restrictions were derided as seeking a “quick fix”.<br><br>Restrictions as ineffective and ‘nannyist’<br><br>Here, advertising restrictions were deemed ill-conceived and ineffective. This was often taken to be self-evident, with no argument advanced. Where explanation was offered, the policy was dismissed as poorly-targeted and statements asserted that alcohol advertising does not affect consumption and that consumption was more proximally influenced by other factors, like price. Such framing predicted that that the policy would be automatically rejected by the public as an example of the “nanny state” needlessly interfering with people’s

Study	Geography	What policy is the AI attempting to influence?	Tactics used	Arguments used
				choices. In keeping with this assertion, members of the public often stated that the government was too bound by vested interests or political donations to even consider it, regardless of whether they personally supported or opposed the policy.
Giesbrecht, 2000 [12]	North America	1) Canada: 1995 warning label proposal.<br>2) USA: Sensible Advertising and Family Education (SAFE) Act, a US bill requiring health warnings in alcohol ads (early 1990s)	2) When broadcast and alcohol industry groups realized the imminence of a vote, they sought a compromise by offering to run a public awareness campaign.	1) The alcohol industry argued that research conducted elsewhere did not demonstrate effectiveness in reducing problems and that other methods were more appropriate. Producers also complained about the high cost of changing labels.
Hope, 2006 [13]	Ireland	Alcohol advertising regulations (early 2000s)	The drinks industry rejected some of the most effective policy measures, while calling for more education. The Drinks Industry Group of Ireland submitted a minority report that clearly illustrated their opposition to effective alcohol policy measures. In essence	One hour after the Ministerial launch in Government buildings, the Drinks Industry Group of Ireland (DIGI) had their own "Press Launch" ... The DIGI press release went on

Study	Geography	What policy is the AI attempting to influence?	Tactics used	Arguments used
			they<br><br>• Questioned the scientific evidence<br>• Opposed reducing overall consumption<br>• Opposed increasing alcohol taxes<br>• Opposed lowering the BAC level<br>• Favoured more educational programmes<br><br>One hour after the Ministerial launch in Government buildings, the Drinks Industry Group of Ireland (DIGI) had their own "Press Launch" ... The DIGI press release went on to say ...<br><br>The alcohol industry continued to lobby against the public health approach and in particular ... the proposed legislation on marketing restrictions to protect children, both measures that would impact on industry profits.<br><br>An insight into the drinks industry approach can be seen in a letter sent from the Managing Director of Diageo Ireland to the Minister for Health and Children and copied to the Taoiseach (Prime Minister) and Tanaiste (Deputy Prime Minister). While the letter was ostensibly to complain regarding	to say ...Regarding the STFA recommendation on alcohol advertising, which called for enactment without delay of the alcohol marketing legislation to protect children, the DIGI stated that "advertising has little effect on overall alcohol consumption but rather is focused on promoting the industry's brands amongst existing consumers"<br><br>An insight into the drinks industry approach can be seen in a letter sent from the Managing Director of Diageo Ireland to the Minister for Health and Children and copied to the Taoiseach (Prime Minister) and Tanaiste (Deputy Prime Minister). While the letter was ostensibly to complain regarding comments made by the Minister for health and Children about Diageo's sports sponsorship,

Study	Geography	What policy is the AI attempting to influence?	Tactics used	Arguments used
			comments made by the Minister for health and Children about Diageo's sports sponsorship, the letter also stated that: "Diageo has welcomed the recent alcohol harm reduction strategy published by the British Prime Minister's Office. It seeks a partnership across government ... We believe such an approach should be considered in Ireland as opposed to what we perceive as the one-dimensional approach currently being pursued."<br><br>Non-compliance with the voluntary industry codes had already been raised by some industry members. A complaint with two TV alcohol advertisements that they claimed contravened both the industry code and the TV code, as outlined in a letter to the Minister for Health and Children, met with no satisfaction over a two-year period. The wider Drinks Industry Group of Ireland acknowledged to the Department of Health and Children that there was justifiable concern at the 'slippage' which had occurred in relation to the implementation of the advertising code and suggested that the new Advertising code (ASAI) was a solid base to move forward. However, the 'new' code	the letter also stated that: "Diageo has welcomed the recent alcohol harm reduction strategy published by the British Prime Minister's Office. It seeks a partnership across government ... We believe such an approach should be considered in Ireland as opposed to what we perceive as the one-dimensional approach currently being pursued."<br><br>Non-compliance with the voluntary industry codes had already been raised by some industry members. A complaint with two TV alcohol advertisements that they claimed contravened both the industry code and the TV code, as outlined in a letter to the Minister for Health and Children, met with no satisfaction over a two-year period. The wider Drinks Industry Group of Ireland

Study	Geography	What policy is the AI attempting to influence?	Tactics used	Arguments used
			retained the clause, “advertisements should not be directed at minors or in any way encourage them to start drinking”, which is extremely difficult to prove thus making it inoperable.<br><br>The drinks industry and advertising industries met the Minister within weeks to explain their efforts to improve self-regulation. In May 2003, the Taoiseach speaking at the European Brewery Convention in Dublin, outlined the proposed alcohol marketing legislation to protect young people. It included restricting alcohol advertising on public transport, in youth centres and at sporting events where young people are participants. He also indicated the legislation would restrict alcohol advertising both on TV with a watershed of 22.00 hours and in cinemas and that all promotional material would carry a health warning about the risks associated with alcohol....The Minister for Health and Children was critical of the sponsorship by Diageo of the Guinness All Ireland Hurling Championship, a popular national game, as sending out the wrong message to a society attempting to curb alcohol abuse and suggested that the GAA	acknowledged to the Department of Health and Children that there was justifiable concern at the ‘slippage’ which had occurred in relation to the implementation of the advertising code and suggested that the new Advertising code (ASAI) was a solid base to move forward. However, the ‘new’ code retained the clause, “advertisements should not be directed at minors or in any way encourage them to start drinking”, which is extremely difficult to prove thus making it inoperable.

Study	Geography	What policy is the AI attempting to influence?	Tactics used	Arguments used
			should show leadership by looking elsewhere for sponsors. The Managing Director of Diageo in a letter to the Minister for Health and Children complained about his comments on the GAA Diageo sponsorship and said "I am both surprised and dismayed that the Guinness sponsorship of the All Ireland Hurling championships has been singled out by you once again as an example of 'undesirable' sponsorship of sports". He rejecting the criticism, saying it was 'totally unfair' and damaging to their business reputation and copied the letter to the Prime Minister and the Deputy Prime Minister.<br><br>During the three-year period (2003 to 2005) a wide range of alcohol industry and media businesses lobbied for self-regulation and extensive discussions took place between the industry and the Department of Health and Children. Meanwhile, in the public arena, there was much talk of the promised legislation but no sign of the legislation. The second STFA Report was published in September 2004 and called for the immediate enactment of the proposed legislation restricting alcohol marketing to	

Study	Geography	What policy is the AI attempting to influence?	Tactics used	Arguments used
			protect children..... In December 2004, a new Voluntary Code on alcohol advertising was announced by the industry with the apparent approval of the Department of Health and Children	
House of Commons: Health Committee: Alcohol, 2010 [14]	UK	General marketing	Q308 - Are you saying that Tesco now puts health awareness information in their alcohol aisle?<br><br>Mr Beadles: Can I step in and say that the Wine and Spirit Trade Association working with the DrinkAware Trust, which is the industry-funded charity, developed point of sale materials which reflected the Chief Medical Officer's health advice and also gave sensible drinking tips for the industry. They were produced towards September and have rolled out in a number of stores. They are not in every store yet, there is no doubt about that, but there are a number of store groups that have developed them. It is an ongoing process and we will be building it.<br><br>Q314: Do you think it is all right to promote alcohol, and I am not saying that Tesco does this but a major supermarket did, at the end of a children's clothes aisle	Q314: Do you think it is all right to promote alcohol, and I am not saying that Tesco does this but a major supermarket did, at the end of a children's clothes aisle<br><br>Mr Beadles: I do not believe that it is appropriate to sell alcohol and to market it there. In fact, the industry codes on the subject say that alcohol should not be promoted alongside anything that would appeal to children. There will always be instances where store managers get it wrong, and we have dealt with a number of those over the past six months where consumers have complained to us that they think a product has been inappropriately placed, and in

Study	Geography	What policy is the AI attempting to influence?	Tactics used	Arguments used
			Mr Beadles: I do not believe that it is appropriate to sell alcohol and to market it there. In fact, the industry codes on the subject say that alcohol should not be promoted alongside anything that would appeal to children. There will always be instances where store managers get it wrong, and we have dealt with a number of those over the past six months where consumers have complained to us that they think a product has been inappropriately placed, and in all instances we have stepped in and the retailer has removed and changed the product location.<br><br>Q388 : The other Jeremy, in relation to the Sheffield report.<br><br>Mr Beadles: We take the same view, and we think there are other economic studies by CeBR and Oxford Economics that I think should be taken into account by this committee. We certainly take the view that the people who misuse alcohol are the least responsive to price changes. We do not argue at all that there is a relationship between price and consumption, but we do think that there is a lot of evidence to	all instances we have stepped in and the retailer has removed and changed the product location.<br><br>Q385 : Minimum pricing. This is for Everyone who wants to come in on this. The Sheffield report on minimum pricing, especially in relation to elasticity of demand for alcohol, Jeremy Blood: do you accept the findings of the Sheffield report? Would you be in favour of minimum pricing?<br><br>Mr Blood: We are not in favour of minimum pricing. I have read the Sheffield report. It draws conclusions about affordability and price. We accept some of the conclusions. Other independent economic advice draws slightly different conclusions from it. As with all research, there is a range of conclusions that can be drawn

Study	Geography	What policy is the AI attempting to influence?	Tactics used	Arguments used
			suggest that there is not a direct link between price and alcohol misuse.<br><br>Q814: I imagine you are using these new media more and more. Is that growing?<br><br>Mr Fennell: Yes.<br><br>Q815: That is going to be the case in the future?<br><br>Mr Fennell: Commensurate with how consumers are changing their behaviour.<br><br>Mr Oak: With regard to new media, in terms of advertising and advertising on sites, specifically we only advertise and the rules are that we can only advertise on sites where 75% of the audience is over 18. That is independently verified. For the sites that we have advertised Lambrini on, a minimum of 88% of the consumers are over 18.<br><br>Q816: That means 25% are under.<br><br>Mr Oak: I accept that point.<br><br>Q817: There is always going to be an overlap, is there not?	from what is a complex set of data. Why do we, in principle, not support minimum pricing? We believe that where misuse is happening and where people are drinking more than is good for them or using alcohol in the wrong way, those are the people that will not change their behaviour if you apply minimum pricing, they will carry on misusing, and you will not address the proper concerns that society has got about the misuse of alcohol through that blanket approach<br><br>Q388 : The other Jeremy, in relation to the Sheffield report.<br><br>Mr Beadles: We take the same view, and we think there are other economic studies by CeBR and Oxford Economics that I think should be taken into account by this committee. We certainly take the view that the people who

Study	Geography	What policy is the AI attempting to influence?	Tactics used	Arguments used
			Mr Oak: Yes.<br><br>Mr Fennell: That is why we need multiple safeguards.<br><br>Ms Carter: Today we have touched on the challenge that all age related categories have and I do not think this is just the UK. The internet is global. Potentially, somebody could be sat in a living room in America and set up a website in the space of five minutes. That is why we created our own digital code two years ago in the absence of anything else. There has been some good work going forward since then but we would certainly welcome any more help in that area.<br><br>Q818: Mr Fennell, in what way does the Diageo code which I have not seen but which has been spoken of considerably this morning differ at all from the ICAP codes on advertising for alcohol?<br><br>Mr Fennell: It is consistent. The code in the exact form of words is a consistent code for Diageo around the world. The first provision in our code is to make sure that we comply with local regulation. The form of words and	misuse alcohol are the least responsive to price changes. We do not argue at all that there is a relationship between price and consumption, but we do think that there is a lot of evidence to suggest that there is not a direct link between price and alcohol misuse.<br><br>Q418: Mr Beadles can start with this but it is really for everyone. For quite a long time now since there has been concern about excessive alcohol consumption, the trade has been interested in voluntary arrangements and voluntary agreements, saying that they want to do something about it. You will all be familiar with the recent KPMG evaluation of voluntary agreements, and they showed extensive breaches of the voluntary code. Why do you think these voluntary

Study	Geography	What policy is the AI attempting to influence?	Tactics used	Arguments used
			the meaning you can draw from them is consistent on every item<br><br>**WRITTEN EVIDENCE**<br><br>**Drinkaware Trust**<br><br>The Drinkaware Trust (Drinkaware) is an independent charity established in January 2007 as a UK wide, public-facing body with the objective of positively changing public behaviour and the national drinking culture to help reduce alcohol misuse and minimise alcohol-related harm, funded by voluntary donations from across the alcohol industry.<br><br>There is continued debate about the most effective methods of reducing excessive alcohol consumption. Legislation, regulation, self-regulation and enforcement all have an important part to play. However, education should always have a central role.<br><br>**National Association of Cider Makers**<br><br>It is misplaced to focus on the availability and	approaches fail?<br><br>Mr Beadles: I do not think they fail, firstly. I think KPMG actually showed a lot of very good practice. I think the issue with a totally voluntary approach is that it only applies to the people who are in and sign up to it. When we looked at the businesses from an off-trade perspective, which, therefore, would fall within our membership and the producer side and their compliance, their compliance level was very high, but when you are trying to take that voluntary approach down to small independent businesses and people who are genuinely not interested in this stuff, then it is very tricky. I think a voluntary approach has an advantage. It tends to be faster and it gets to the core of big business quicker, but it is not going to ever get overall

Study	Geography	What policy is the AI attempting to influence?	Tactics used	Arguments used
			affordability (price and promotion) of alcohol as the sole and root cause of misuse. The real drivers behind harmful drinking, binge drinking behavior and under 18's alcohol misuse tend to get overlooked as a consequence. This means adopting or calling for a combination of long-term measures such as improving education, awareness campaigns, etc<br><br>NACM firmly believes that any policy considerations, by the Government, to address the specific misuse of alcohol by problem drinkers, should be:<br><br>1. Based on robust evidence (ensuring that studies are peer reviewed) that is relevant to equivalent environments, as opposed to deploying convenient data to fit an argument.<br><br>A key point that comes across in the School of Health and Related Research at Sheffeld University (ScHARR) Study on "Price, Promotion and Harm" is that very little research has been conducted in the UK that throws light on individual behaviour with regard promotional activity of alcoholic	coverage. Having said that, there is lots of legislation that is not complied with by lots of business as well. So I think voluntary approaches have a hugely important role to play, but you have always got to recognise that there will be some people who sit outside them.<br><br>Q559Dr Taylor: As the Portman Group is funded by the drinks industry I admit a conflict of interest in that I have a very small number of Diageo shares. I shall not enlarge on that.Mr Lewis, you are quite an experienced witness; you have appeared before us in other inquiries in the not too distant past. You mentioned the effctiveness of the Drinkaware Trust which is to promote sensible drinking. Could it be that the Portman Group is looking to reduce your funding because you are being

Study	Geography	What policy is the AI attempting to influence?	Tactics used	Arguments used
			drinks and harms. A significant amount of the material ScHARR draws on derives from the USA. A basic sociological critique of the ScHARR report would immediately pick up on the "situational relevance" of those studies to the UK.<br><br>The Sheffield University review failed to provide compelling evidence that would warrant the Government to introduce controls on promotions and advertising. The review cited publications and references making causal links but some of these documents have not been peer reviewed. NACM reiterates its position that evidence should be robust.<br><br>**Diageo**<br><br>Diageo has proposed to Government the creation of three important new initiatives:<br><br>— a new mandatory code on retail promotions, within a framework of co-regulation, under the auspices of The Portman Group;<br><br>— a co-regulatory approach on mandatory labelling of alcohol, also under the auspices	effective in promoting sensible drinking which is what we want but not what the drinks industry wants?<br><br>Mr Lewis: The Portman Group has been the most stalwart group in funding the trust.<br><br>Q560 Dr Taylor: So far?<br><br>Mr Lewis: Yes.<br><br>Q561 Dr Taylor: But you are so good that they will remove it?<br><br>Mr Lewis: The Portman Trust has made no suggestion that it will reduce its commitment. We always hope that it may increase it. An important point to dwell upon for a moment is that the trust is an independent organisation with a very specific remit which is to provide information and education. It is evidence-based so that the consumers of alcohol and other interested

Study	Geography	What policy is the AI attempting to influence?	Tactics used	Arguments used
			of The Portman Group; and<br><br>— an industry-wide significant new social marketing partnership, aiming towards changes in attitudes and behaviour among certain target groups in the UK.<br><br>**Scotch Whisky Association and the Gin & Vodka Association**<br><br>We believe in a targeted approach aimed at "at risk" populations. We consider that working in partnership with all stakeholders is fundamental to tackling the issue and that education coupled with robust enforcement and responsible marketing and promotion are key elements to creating and underpinning a culture of responsible drinking.<br><br>Education in its widest sense is fundamental to a better appreciation of the risks associated with the misuse of alcohol. It can be used to target a range of problem drinkers: Alcohol education should be introduced in schools at an early and formative age and be a compulsory part of the curriculum and we welcome the DCSF's	parties can make informed judgments about how they use it. The question sometimes raised is whether that is possible when the funding comes from the alcohol industry and we have on our board people who are employed by it, but the trust does defend its independence with great rigour. We view the presence of people on its board from the alcohol industry as helpful because it brings a considerable amount of expertise, but they are in a minority. When they are on the board they are there to represent the interests of the trust, not their parent organisations.<br><br>Q569:Mr Poley, in the past I have had numerous discussions with representatives of the Portman Group. They argue that the drinks industry engage in

Study	Geography	What policy is the AI attempting to influence?	Tactics used	Arguments used
			recent commitment to introduce this measure. Children should be given sufficient information about alcohol to be able to discuss with their parents issues around alcohol consumption (much as children have done in relation to smoking or recycling). As well as running individual social responsibility programmes and supporting the Drinkaware Trust, an independent charity set up to help consumers make informed decisions about alcohol, the alcohol industry has responded to the Government's call to use marketing expertise to develop a campaign to tackle alcohol-misuse—particularly among young people age 18–24. Alcohol education should be reinforced in adult life through effective Government multi-media campaigns, supported by the industry and other stakeholders. Better communication of responsible drinking messages to the adult drinking population through measures, such as on-label messaging and advertising can also contribute in this area.<br><br>**Portman Group**<br><br>We consider, however, that better	advertising in order to encourage the consumption of one brand rather than another but then they deny that it encourages an increase in alcohol consumption overall. I just think it is very unlikely that that is true, but what do you think of it in the context of what we have just been talking about?<br><br>Mr Poley: ... To turn to the question of the advertising effect, there is a wealth of evidence to demonstrate that the predominant effect of advertising is to cause brand switching. Even the report from the University of Sheffield commissioned by the Department of Health last year found that the evidence for the effect of alcohol advertising upon consumption was relatively weak. At best it will have a slight effect.

Study	Geography	What policy is the AI attempting to influence?	Tactics used	Arguments used
			education, providing early advice and support to problem drinkers... The industry has an obligation to market its products responsibly so as not to encourage their misuse. It has a further obligation to use its reasonable endeavours to educate consumers and proactively encourage responsible drinking.<br><br>We are committed to regularly reviewing our Code rules through public consultation involving key stakeholders to ensure that the Code evolves and that it is indeed offering a proper balance between protecting the public from irresponsible drinks producer marketing while at the same time allowing companies reasonable commercial marketing freedoms.<br><br>Educational Initiatives: The Portman Group and its member companies have pioneered a number of inspiring alcohol responsibility initiatives which include: Drinkaware website, labelling, Drinkaware Trust<br><br>Education and campaigning can successfully challenge potentially harmful perceptions surrounding drinking. This is why social	Q576: What is the attitude of the witnesses to health warnings on alcohol products?<br><br>Mr Poley: All of our member companies are committed to putting certain information on their drinks packaging; that is to say, they have information that they put on voluntarily about the number of alcohol units they contain. They also put on the Drinkaware Trust website address and they will also have a responsible drinking message such as "Please drink responsibly". I do not believe it is necessarily appropriate to have a health warning on a drink of alcohol. Alcohol is not like cigarettes; it is capable of being misused but when drunk in moderation it is perfectly compatible with a healthy lifestyle. For certain groups when drunk in moderation it has health advantages. To put on a

Study	Geography	What policy is the AI attempting to influence?	Tactics used	Arguments used
			marketing is of fundamental importance and explains why the Portman Group is such an ardent supporter of the Drinkaware Trust<br><br>**Scottish and Newcastle**<br><br>We strongly believe that industry can be a valuable partner in tackling alcohol misuse, by marketing its products responsibly, providing consumers with information—demonstrated by our full compliance with the voluntary label—and delivering education and responsible drinking programmes.<br><br>S&N UK believes that industry self-regulation can effectively ensure alcoholic drinks are advertised and promoted responsibly. We are a founding member of the Portman Group, a self-regulatory organisation that encourages and challenges the industry to promote its products responsibly. The recent KMPG report commissioned by the Department of Health recognised the Portman Group had delivered considerable improvement in alcohol marketing. The Portman Group's Marketing Code of Practice was introduced in 1996 and is the basis of	soundbyte in the form of a health warning label seems to me to be an unbalanced way to convey the complex information about the health effects of drinking<br><br>Q780: I have a question to all of you. You will be aware that the Royal College of Physicians estimate that the number of deaths caused by alcohol misuse in the UK is about 40,000 a year and with the social cost put at billions of pounds. Last week the British Medical Association called for a complete ban on alcohol advertising and the introduction of minimum unit pricing. Do you think the time has come to restrict the availability, price and promotion of alcohol?<br><br>Ms Carter: One of the first things we would like to pick up on, particularly with regard to

Study	Geography	What policy is the AI attempting to influence?	Tactics used	Arguments used
			S&N UK's Responsible Marketing Policy. It provides guidance to everyone involved in marketing and selling our products to ensure our commercial communications do not contribute to excessive consumption or abuse. We always ensure that our policy goes beyond the requirements of industry voluntary codes.<br><br>S&N UK welcomes more investment in education and information on sensible drinking. We believe the industry has a vital role to play in providing consumers with relevant information about their products. Originally created by the Portman Group, the Drinkaware Trust is an educational charity that aims to positively change the UK drinking culture. It is a unique partnership between industry and the voluntary and public health sectors. The Trust receives its funding from voluntary donations by the drinks industry.<br><br>**Wine and Spirits Trade Association**<br><br>Studies have shown the importance of education from a young age and from a variety of sources. Positive messages must	an advertising ban, one of our overriding thoughts would be that an alcohol advertising ban would not actually stop people going out to pubs on a Saturday night, having alcohol with their barbeques or dinner parties. So I think one has to look at the wider marketing mix of alcohol. Additionally there are other alcohol categories that are not big alcohol advertising spenders but actually see strong growth. The wine industry would be an industry in which we have seen some phenomenal growth in the last 15 years but in relative terms has actually been a small alcohol advertising spender. So you have seen that there are lots of different dynamics in the marketing mix that could affect that. So we would say that you would have to look at all the elements together and an advertising ban would not

Study	Geography	What policy is the AI attempting to influence?	Tactics used	Arguments used
			not only come from teachers and public health campaigns but from friends and family. Family attitudes have also been shown to be important in prevalence of drinking.<br><br>Increasing consumers' knowledge and appreciation of alcoholic products, through tastings and education courses also plays an important role in ensuring people drinks for the right reasons and do so in a healthy way. Wines and spirits can be a fascinating area of interest and teaching consumers to appreciate this is a more effective way of encouraging mature attitudes to alcohol than by demonising it through measures such as separate checkouts and tobacco style health warnings.<br><br>Providing information to the consumer is a key part of changing culture and to this end, the drinks industry fund the Drinkaware Trust<br><br>**SABMiller**<br><br>SABMiller is proactive in providing consumers with accurate and balanced	really help. We would also be worried about the potential unintended consequences of that and might it push some producers to shift some of that money in their competition to gain volume share into more price activity, not necessarily in terms of depth of deal but maybe frequency of it.<br><br>Mr Oak: Adding to what Deborah has said, ultimately the advertising industry is heavily regulated already. Price and advertising are just but two elements within that and if you ban advertising you then have to look at all the other elements like PR, sponsorship, etcetera and there is no guarantee and no evidence to state that if you ban advertising you will prevent misuse, which is ultimately what we are all here talking about today. I think in terms of minimum pricing, yet again it

Study	Geography	What policy is the AI attempting to influence?	Tactics used	Arguments used
			information about alcohol through our innovative website, www.TalkingAlcohol.com. This award-winning website, the first of its kind from a major alcohol producer, describes in detail the health and social considerations of drinking alcohol such cancer, liver disease, and stroke among others are all discussed in a factual, balanced manner.... Elsewhere, industry has worked to provide relevant and factual information to consumers in accessible means such as through initiatives undertaken by the Drinkaware Trust of which SABMiller was the first corporate member.<br><br>SABMiller recently commissioned Centre for Economics and Business Research (CEBR) to evaluate the ScHARR review of alcohol pricing and promotion effects on consumption and harm. The CEBR research found that... minimum pricing is an incredibly blunt instrument which imposes significant costs across large sections of society, whilst having very limited benefits in terms of curbing the excesses of the minority.	has been clearly documented that pricing can help reduce per capita consumption—I think that came out in the Sheffield Report, but it did not say that it could help reduce misuse. One of the concerns I particularly have with regard to Lambrini is that Lambrini is consumed by everyday women, hardworking women and a lot of those women are on a low budget—62% of them earn £17,000 a year and I would have the concern that minimum pricing could make alcohol become quite an elitist product, and that is not the case and should not be the case for people drinking it responsibly.<br><br>Q782: You have all said that banning advertising would not have the effect in terms of binge drinkers but what effect would it have on sales? What lessons can be learned from

Study	Geography	What policy is the AI attempting to influence?	Tactics used	Arguments used
				the restrictions of alcohol advertising in countries like France with their Loi Evin? Presumably you have looked at that?<br><br>Mr Fennell: Yes, we have and we should learn from our experience from all around the world. The evidence suggests that advertising does not have an effect on total consumption whether it is present or not. The trends when legislation has changed have broadly stayed the same. Indeed, the total alcohol market in this country has been flat or declining for years. Our job and the role of our advertising place is to take business off each other. It is a zero sum game. All of our advertising should promote, as Simon said, responsible consumption and it is intended to win market share. As a result Smirnoff only constitutes 3% of the market

Study	Geography	What policy is the AI attempting to influence?	Tactics used	Arguments used
				here in the UK so we see a considerable growth for our brands without any growth in the market.<br><br>Q795 :A quick point on the Lambrini. If you walk round almost any town it seems to be quite a favourite of underage teenage girls, so would not minimum pricing help reduce that market? You probably do not make an assessment of what teenagers drink because they are not supposed to be drinking.<br><br>Mr Oak: We do not support or promote drinking to underage girls. For many of those people that you described that may be drinking alcohol very often that alcohol has been purchased by someone else. I do not believe in that sense that you are talking to the consumer or the purchaser of the alcohol, so price has a different bearing in

Study	Geography	What policy is the AI attempting to influence?	Tactics used	Arguments used
				that instance<br><br>Q805: Let us focus on the misusers, which is absolutely right. We have to focus on the misusers. In your submission you have listed who they are. How do we focus on them? How do we get at them? How do we control them?<br><br>Mr Fennell: That is a great question.<br><br>Q806: Yes. Have you an answer?<br><br>Mr Fennell: ... I also think we should enforce rigorously the high level of regulation that we already have in the UK...... We should enforce that more firmly. I wish we could ensure that we give the police the powers to sort out social disorder when it happens immediately and quickly and deal with it. It is a combination

Study	Geography	What policy is the AI attempting to influence?	Tactics used	Arguments used
				of enforcing the regulations that we have, education and ensuring that our code, to which we subscribe, is rigorously employed internally and has the right backstop of independent measures associated with it.<br><br>Q814: I imagine you are using these new media more and more. Is that growing?<br><br>Mr Fennell: Yes.<br><br>Q815: That is going to be the case in the future?<br><br>Mr Fennell: Commensurate with how consumers are changing their behaviour.<br><br>Mr Oak: With regard to new media, in terms of advertising and advertising on sites, specifically we only advertise and the rules are that we can only advertise on sites where 75% of the audience is over 18.

Study	Geography	What policy is the AI attempting to influence?	Tactics used	Arguments used
				That is independently verified. For the sites that we have advertised Lambrini on, a minimum of 88% of the consumers are over 18.<br><br>Q816: That means 25% are under.<br><br>Mr Oak: I accept that point.<br><br>Q817: There is always going to be an overlap, is there not?<br><br>Mr Oak: Yes.<br><br>Mr Fennell: That is why we need multiple safeguards.<br><br>**WRITTEN EVIDENCE**<br><br>**Diageo:**<br><br>The first Online Reputation report was written on 16 November 2007...of over 500 Facebook sites that mention the word Smirnoff, only 36

Study	Geography	What policy is the AI attempting to influence?	Tactics used	Arguments used
				have more than 100 members. The total reach of these 36 sites is c. 15k people (and Facebook has over 3 million users in GB).<br><br>In producing the report, Splendid has looked at each Facebook site with references to Smirnoff and the extent to which the material contained therein would have breached Diageo's Marketing Code. However, unlike Diageo generated online content which is subject to the Diageo Marketing Code and approval process, dealing with problematic user generated content on third party Internet sites is, by the very nature of the internet, almost impossible given the ease with which such content can be spread and replicated on different sites and forums and the resource that would be required to try and continuously monitor and

Study	Geography	What policy is the AI attempting to influence?	Tactics used	Arguments used
				police content.<br><br>However, one action that we believe has helped in this area is the creation of our own official Smirnoff Facebook page. This currently numbers over 61,000 members and we are able to monitor and remove content from this page that would breach the Diageo Marketing Code. A further development, which we believe should also assist in reducing, at least on Facebook, the amount of inappropriate content, is that Facebook has recently asked Diageo for the URL details of all official Smirnoff Facebook pages so that all other pages can be closed down by them. We would encourage site operators to adopt similar strategies. Our primary means of enhancing the online reputation of our brands, is to actively create positive

Study	Geography	What policy is the AI attempting to influence?	Tactics used	Arguments used
				consumer experiences that will result in positive posts. The kinds of action that we have undertaken in this area are encouraging consumers to attend Smirnoff events around the country, at which responsible consumption messages play a key part, and then subsequently post their comments, pictures and videos online on our official Facebook page, and hosting briefings for bloggers and providing them with exclusive information that they may choose to post in their blogs.<br><br>**Portman Group**<br><br>the Committee seems to be under the impression that there is a "regulatory gap" in respect of on-line marketing and sponsorship... I recognise that the regulatory system is complex but the fact is that all drinks producers' marketing

Study	Geography	What policy is the AI attempting to influence?	Tactics used	Arguments used
				activity is subject to the same strict standards of regulation; there are no “gaps”.<br><br>For historical reasons, three regulators are involved: Ofcom, the Advertising Standards Authority (ASA) and the Portman Group. The different regulatory systems, however, while they operate independently of one another, adopt similar standards and complement one another to ensure strict supervision of all drinks producer marketing activity... the Portman Group seeks to regulate any marketing which is not otherwise regulated by Ofcom/ASA<br><br>**Constellation Europe**<br><br>Constellation believes that the majority of the public in England, who consume wine, do so on a responsible and

Study	Geography	What policy is the AI attempting to influence?	Tactics used	Arguments used
				enjoyable basis.<br><br>Wine suppliers like Constellation consider that they can play a market based role in encouraging a more responsible approach to alcohol consumption among the public, where this is desired.<br><br>Such a strategy entails investing in the category through brand building and consumer education in order to encourage consumers to trade up to better wines and with it drive a better drinking experience.<br><br>This move to adopt a different drinking behaviour would also have a positive impact on consumption and misuse. Similar strategies have been successful in the United States.<br><br>Constellation does not support

Study	Geography	What policy is the AI attempting to influence?	Tactics used	Arguments used
				moves to further ban advertising and promotion of wine, rather to use marketing strategies to promote a more responsible approach to alcohol consumption.<br><br>**Drinkaware Trust**<br><br>The Drinkaware Trust (Drinkaware) is an independent charity established in January 2007 as a UK wide, public-facing body with the objective of positively changing public behaviour and the national drinking culture to help reduce alcohol misuse and minimise alcohol-related harm, funded by voluntary donations from across the alcohol industry.<br><br>There is continued debate about the most effective methods of reducing excessive alcohol consumption. Legislation, regulation, self-

Study	Geography	What policy is the AI attempting to influence?	Tactics used	Arguments used
				regulation and enforcement all have an important part to play. However, education should always have a central role. At the root of the alcohol misuse problem in the UK is behaviour ingrained in our culture for thousands of years. The public cannot be expected to accept attempts to change these deep-rooted social habits without clear information about the extent of the problem, and how it applies to them.<br><br>**Molson Coors**<br><br>It is our view that our marketing and advertising practices, whilst constantly under review, demonstrate our determination to behave in a responsible manner.<br><br>We do not know definitively whether the introduction of minimum pricing in the United

Study	Geography	What policy is the AI attempting to influence?	Tactics used	Arguments used
				Kingdom is the right way to go. As Committee Chairman, you will be well aware of the very diverse views on this subject. Price is, in our opinion, certainly not a sole or decisive factor in addressing alcohol abuse . Certainly there are no single measures that, by themselves, will solve the problem.<br><br>... while we generally do not support Government price controls, we believe that social reference pricing may be helpful in the exceptional case of extremely low prices in the UK. To this end, I have made a number of public comments suggesting that a more detailed examination of the issue of minimum pricing would be desirable and that we would wish to take an active part in such consideration.

Study	Geography	What policy is the AI attempting to influence?	Tactics used	Arguments used
				Molson Coors is a family brewer, I am a family man, behaving properly is an integral part of our collective and my personal commitment to operating to the highest standards. My Company and I are proud of our product and will champion it wherever we can. Part of that championing is to make sure that we actively seek new ways of ensuring that our product is always consumed responsibly. I am very pleased to be able to take this opportunity, on behalf of my Company, to offer every assistance to your Committee in its work.<br><br>**National Association of Cider Makers**<br><br>A review of published reports in the public domain produced by the UK Government and other bodies, quite clearly demonstrates that no one

Study	Geography	What policy is the AI attempting to influence?	Tactics used	Arguments used
				alcoholic drink is responsible for alcohol misuse—misuse is caused by certain drinkers who clearly misuse alcohol and by some under 18s who are clearly breaking the law. This therefore is not a problem about problem drinks but about problem drinkers.<br><br>It is misplaced to focus on the availability and affordability (price and promotion) of alcohol as the sole and root cause of misuse. The real drivers behind harmful drinking, binge drinking behavior and under 18's alcohol misuse tend to get overlooked as a consequence. This means adopting or calling for a combination of long-term measures such as improving education, awareness campaigns, etc and short-term measures which can also be effective such as enforcing the

Study	Geography	What policy is the AI attempting to influence?	Tactics used	Arguments used
				legislation that already exists.<br><br>NACM acknowledges that Government has committed resources to initiatives in this area as indeed has industry. NACM is a supporter of the Drinkaware Trust and of “Project 10” a £100 million (over five years) industry education initiative.<br><br>It is important to bear in mind that the distinction must be made and maintained to ensure that whatever means are introduced they are targeted at preventing harmful drinking without punishing the majority of people who drink responsibly. The industry is concerned that the introduction of measures that have a broader impact and go beyond targeting problem drinkers will bring with it unintended consequences.

Study	Geography	What policy is the AI attempting to influence?	Tactics used	Arguments used
				To this end industry has a legitimate role to play in working with Government in reaching solutions.<br><br>NACM firmly believes that any policy considerations, by the Government, to address the specific misuse of alcohol by problem drinkers, should be:<br><br>1. Based on robust evidence (ensuring that studies are peer reviewed) that is relevant to equivalent environments, as opposed to deploying convenient data to fit an argument.<br><br>2. Given that the majority of the public drink sensibly and that alcohol is misused by a minority of drinkers, general population measures such as increasing taxes or other means of raising prices (curbing promotions, introducing minimum pricing

Study	Geography	What policy is the AI attempting to influence?	Tactics used	Arguments used
				etc.) are not the appropriate means for tackling misuse—it penalises the majority of sensible drinkers without necessarily dealing with alcohol misuse. People's lives are already being negatively impacted by problem drinkers and it seems ironic that this negative impact should be doubly visited upon them by having to endure restrictions, inconvenience, and ultimately higher prices, as set out in the consultation document, to deal with the problem drinkers.<br><br>3. Dealt with, in the many instances of misuse, by better/more effective enforcement. No new legislation is required. NACM believes that the Government should focus on maximising the effective use of existing legislation to target problem drinkers and that it should avoid using one-size-fits-all

Study	Geography	What policy is the AI attempting to influence?	Tactics used	Arguments used
				measures that just punish everyone.<br><br>4. There is a need to ensure that measures proposed do not in fact work against the Government's overarching objective of reducing alcohol related harm.<br><br>A key point that comes across in the School of Health and Related Research at Sheffeld University (ScHARR) Study on "Price, Promotion and Harm" is that very little research has been conducted in the UK that throws light on individual behaviour with regard promotional activity of alcoholic drinks and harms. A significant amount of the material ScHARR draws on derives from the USA. A basic sociological critique of the ScHARR report would immediately pick up on the "situational relevance" of

Study	Geography	What policy is the AI attempting to influence?	Tactics used	Arguments used
				those studies to the UK.<br><br>Notwithstanding these short-comings ScHARR has failed to recognise that promotional activity provides a direct and more effective way to introduce consumers to new products and product variants. Restricting promotions will inhibit new product introductions. The average strength of cider in the UK has reduced. There are also plans to introduce into the UK market significantly lower strength cider but before the product is put on sale more widely it is being trialed/promoted in a limited number of retail outlets. However, without the ability to promote such new products, producers will not be able to introduce them to the public at large. Of concern is that inevitably the market will stagnate and will become

Study	Geography	What policy is the AI attempting to influence?	Tactics used	Arguments used
				characterized by lower quality and cheaper products.<br><br>Advertising is already strictly regulated. NACM does not believe that any further restrictions on advertising will address any particular misuse issues. The consequences of further restrictions will be to shut down a further avenue for bringing to market newer and better quality products.<br><br>The Sheffield University review failed to provide compelling evidence that would warrant the Government to introduce controls on promotions and advertising. The review cited publications and references making causal links but some of these documents have not been peer reviewed. NACM reiterates its position that evidence should be robust.<br><br>As mentioned above members

Study	Geography	What policy is the AI attempting to influence?	Tactics used	Arguments used
				of the NACM are introducing the labelling scheme information on bottles and cans. However, this can only be done with planned label changes over a period of time to avoid writing off (and additionally creating its own waste issues) £ms of packaging materials.<br><br>NACM is aware that the Government is holding legislation in reserve to secure a greater uptake of the labelling scheme information. If this approach were to be actively pursued it would halt, in its tracks, any further voluntary introduction of the key components of the labelling scheme because it would introduce uncertainty as to what would be specifically required to be included on cans and bottles re exact wording, use or non use of the pregnancy logo, dimensions of

Study	Geography	What policy is the AI attempting to influence?	Tactics used	Arguments used
				characters, positioning of statements etc. The industry would not want to write off two sets of packaging. Furthermore legislation would recognise the industry's legitimate request for a period of 12 to 18 months for transition period to permit existing non complying packaging to be sold through.<br><br>Penalising the industry and the general population is not an appropriate way forward in either seeking to bring about the desired changes in reducing alcohol harm or dealing with alcohol misuse (the problem drinkers).<br><br>Furthermore before any action is considered with regard to promotions and advertising it is vital that research is undertaken that is situationally relevant to the UK to avoid ill-considered policy proposals:

Study	Geography	What policy is the AI attempting to influence?	Tactics used	Arguments used
				proposals that could undermine efforts to promote a cultural change if new products are not provided a route to market.<br><br>To restate, there are no problem drinks, only problem drinkers and therefore measures need to be targeted at these misusers. Furthermore NACM believes that the panoply of powers available to the police and local authorities should be used much more effectively both against individuals who misuse alcohol and those who willfully seek to break the law in obtaining alcohol underage, as well as against those retailers who sell alcohol irresponsibly.<br><br>**Diageo**<br><br>As a leading premium drinks producer, we recognise that responsible drinking is

Study	Geography	What policy is the AI attempting to influence?	Tactics used	Arguments used
				important both to our business interests and to society's interests, and that we have a role to play in raising awareness and seeking to influence attitudes and behaviour among consumers of our products.<br><br>We believe that alcohol policy should be fair, effective, proportionate, consistent and evidence-based, and that it should not have unintended economic or social consequences. We do not believe that effective alcohol policy automatically or necessarily involves new legislation or regulation.<br><br>Diageo believes that the potential for harm is preventable and that a valuable and sustainable place in society exists for alcohol beverages. Diageo believes that better enforcement of

Study	Geography	What policy is the AI attempting to influence?	Tactics used	Arguments used
				existing laws and regulations and better partnership working can deliver a more responsible drinking culture. This requires the skills, experience and contributions of all stakeholders to be leveraged more effectively. Clarity on their sphere of influence and interaction is key.<br><br>A wide range of stakeholders have a role to play and include: individual consumers... national government... alcohol producers and retailers<br><br>Diageo has proposed to Government the creation of three important new initiatives:<br><br>— a new mandatory code on retail promotions, within a framework of co-regulation, under the auspices of The Portman Group;

Study	Geography	What policy is the AI attempting to influence?	Tactics used	Arguments used
				— a co-regulatory approach on mandatory labelling of alcohol, also under the auspices of The Portman Group; and<br><br>— an industry-wide significant new social marketing partnership, aiming towards changes in attitudes and behaviour among certain target groups in the UK.<br><br>Responsible drinking is also at the heart of our business interests. Our reputation as a business and the reputation of our brands are damaged when our products are misused. We do not want to be targeted as a cause of anti-social behaviour, of drunkenness or of damage to our consumers' health. We understand that governments, regulators and society will rightly act to curtail alcohol misuse where it occurs, but that inappropriate or ineffective legislative and

Study	Geography	What policy is the AI attempting to influence?	Tactics used	Arguments used
				regulatory actions may cause disproportionate damage to our business interests.<br><br>The relationship between individuals and alcohol is ultimately a personal one: individuals decide for themselves if, when, where, how much and how often they drink. But a wide range of stakeholders have a role in influencing that individual decision and enabling the consumer to make informed choices. Clarity on their sphere of influence and interaction is key.<br><br>We have identified three key priorities for responsible drinking within Diageo:<br><br>— set world-class standards for responsible marketing and innovation;<br><br>— combat alcohol misuse,

Study	Geography	What policy is the AI attempting to influence?	Tactics used	Arguments used
				working with others on initiatives to reduce alcohol-related harm; and<br><br>— seek to promote a shared understanding of what it means to drink responsibly. This is underpinned by our use of our marketing insight and skills in an attempt to transform consumers' attitudes to alcohol.<br><br>We hope that the Government will continue to consider co-regulation in delivering a mandatory code as well as respond positively to the other initiatives outlined in this submission, and that other stakeholders will also act, so that we can have a positive, collective effect in raising awareness and shifting attitudes and behaviours on alcohol and reducing alcohol misuse among the specific minority groups most at risk of

Study	Geography	What policy is the AI attempting to influence?	Tactics used	Arguments used
				alcohol-related harm.<br><br>**Scotch Whisky Association and the Gin & Vodka Association**<br><br>The Scotch Whisky Association (SWA) and Gin & Vodka Association (GVA) are committed to working in partnership with Government and other stakeholders to tackle alcohol misuse. It is the minority that misuse alcohol. Adoption of a blanket "one-size" fits all approach does nothing to support the responsible majority—whether consumer or trader.<br><br>We believe in a targeted approach aimed at "at risk" populations. We consider that working in partnership with all stakeholders is fundamental to tackling the issue and that education coupled with robust enforcement and responsible marketing and promotion are

Study	Geography	What policy is the AI attempting to influence?	Tactics used	Arguments used
				key elements to creating and underpinning a culture of responsible drinking.<br><br>We recognise and share the desire to tackle alcohol misuse. Indeed, the SWA and the GVA have been active in promoting responsible drinking for some 40 years. The alcohol industry—alongside other stakeholders—clearly has a role to play in encouraging and promoting the responsible consumption of its products to protect the long-term sustainability of the industry.<br><br>The drinks industry has a long history in promoting responsible drinking... Both Associations and our members are committed to helping foster a step change in cultural attitudes to the consumption of alcohol in the UK, which recognises that responsible, moderate consumption is part

Study	Geography	What policy is the AI attempting to influence?	Tactics used	Arguments used
				of a modern, healthy society and that alcohol misuse is unacceptable.<br><br>Tackling alcohol misuse and achieving cultural change in attitudes to alcohol requires a long-term commitment, close collaboration and a concerted effort by a wide range of public and private stakeholders. It requires tough enforcement of existing alcohol laws, a sound regulatory framework, as well as an innovative approach to social marketing which reaches the right sections of the population. We welcome the government's decision to consult on its alcohol strategy and look forward to responding to the forthcoming consultation on the Government's proposed mandatory code of practice on alcohol promotions.<br><br>The majority of consumers do

Study	Geography	What policy is the AI attempting to influence?	Tactics used	Arguments used
				drink responsibly. We believe that an approach targeting "at risk" populations and potentially harmful contexts and drinking patterns will be most effective.<br><br>We embrace a partnership approach with Government and all stakeholders, focusing action on evidence–based measures. Such an approach breaks down barriers between the various stakeholders, fosters co-operation and allows the different stakeholder groups to share their experiences and build on best practice.<br><br>A fundamental deliverable from producers is responsible marketing and promotion of their brands. Advertising, promotions and sponsorship are a legitimate part of commercial activity in every industry. They play an

Study	Geography	What policy is the AI attempting to influence?	Tactics used	Arguments used
				important part in competition between brands, and are well governed by Codes of Practice/Conduct such as Broadcast Committee of Advertising Practice (BCAP)/Committee of Advertising Practice (CAP) and the Portman Group Code of Practice on the Naming, Packaging and Promotion of Alcohol Drinks. The Code of Practice, which is supported by over 140 companies, is strongly enforced through an independent robust complaints process which includes the power to enforce sanctions for breaches of the Code.<br><br>We welcome the recognition by Government that the majority of retailers and consumers do act responsibly, and that the responsible majority should not be penalised. We believe a clear definition of what is meant by

Study	Geography	What policy is the AI attempting to influence?	Tactics used	Arguments used
				“irresponsible promotion” within different retail environments would be beneficial.<br><br>It is important to recognise that promotions are a very important tool and take many forms (ie on pack promotions such as a free glass or opportunity to enter a competition/prize draw, or opportunity to receive a money off voucher on a new brand when purchasing a bottle).We do not believe such promotions are irresponsible or lead to irresponsible consumption. An issue which has been receiving much attention is price-based promotions. The SWA and GVA are not opposed to appropriate price-based promotions. They are important in contributing towards brand awareness and introducing new products to

Study	Geography	What policy is the AI attempting to influence?	Tactics used	Arguments used
				the market. Without such mechanisms, established brands have an advantage and this can lead to market stagnation. The SWA and GVA would like to make clear our view that we oppose any sort of minimum pricing as a matter of principle, and question whether such a system would be legal under EU and international trade rules.<br><br>**Portman Group**<br><br>The Portman Group (TPG) was set up in 1989 by the UK's leading alcohol producers. Its purpose was to promote sensible drinking; to help prevent alcohol misuse; to encourage responsible marketing; and to foster a balanced understanding of alcohol-related issues.<br><br>A dilemma facing Governments is whether to use alcohol

Study	Geography	What policy is the AI attempting to influence?	Tactics used	Arguments used
				policies to reduce the amount of alcohol that all drinkers consume in order to impact upon harmful drinking even though, by its very design, this approach penalises all drinkers alike. We believe that attempting to tackle problems through reducing per capita consumption (eg through taxation or restrictions on availability) is untargeted and unfair.<br><br>We strongly believe the focus should be on reducing alcohol misuse. It is possible, indeed very probable, that reducing alcohol misuse will actually result in a net decrease in the nation's alcohol consumption but that doesn't mean that reducing overall consumption is an appropriate goal in itself.<br><br>We consider, however, that better education, providing early advice and support to

Study	Geography	What policy is the AI attempting to influence?	Tactics used	Arguments used
				problem drinkers, and a more robust approach to alcohol law enforcement across the UK would deliver further improvements.<br><br>The industry has an obligation to market its products responsibly so as not to encourage their misuse. It has a further obligation to use its reasonable endeavours to educate consumers and proactively encourage responsible drinking.<br><br>Drinks producers are comprehensively regulated by the BCAP/CAP Advertising Codes (overseen by the Advertising Standards Authority) and the Portman Group's Code of Practice on the Naming, Packaging and Promotion of Alcoholic Drinks. These codes (except for the BCAP Advertising Code) are self-regulatory and are

Study	Geography	What policy is the AI attempting to influence?	Tactics used	Arguments used
				therefore sometimes referred to as "voluntary". This term needs to be clarified, however, as it is potentially misleading. The codes are voluntary in so far as the industry has volunteered to impose the restrictions on itself. As is explained below, however, compliance with the codes is mandatory; there is no opt-out for any drinks manufacturer.<br><br>The reason drinks companies advertise is to encourage brand switching. Studies have demonstrated that responsible advertising can have a powerful effect on the type of alcohol people prefer but does not encourage either the onset of drinking or potentially harmful drinking behaviour.<br><br>The Portman Group's Code of Practice applies to the naming and packaging of alcoholic drinks and the promotional

Study	Geography	What policy is the AI attempting to influence?	Tactics used	Arguments used
				activities of drinks producers, including press releases, websites and sponsorship. It ensures that such activities are carried out in a socially responsible way. All complaints made under our Code are heard by an Independent Complaints Panel. This Panel is chaired by Sir Richard Tilt, former Director General of the Prison Service; none of the Panel works in the alcohol industry... Failure to comply with our Code results in a drink being removed from sale... This sanction provides a strong commercial threat to companies, encouraging them to ensure that their marketing is responsible.<br><br>Some commentators claim that the problem of alcohol misuse in the UK is made worse by the price is manipulated to encourage sales. Pricing is, of course, controlled by retailers

Study	Geography	What policy is the AI attempting to influence?	Tactics used	Arguments used
				not producers. Producers are nonetheless interested in the way in which their products are priced and promoted by the retailer. In general, we do not accept that price is the main contributory factor in harmful drinking.<br><br>Price and total consumption are undoubtedly closely related in that price hikes will usually lead to a fall in a nation's consumption. But the effect of price rises on the behaviours of the heaviest drinkers is ambiguous. There are nonetheless some specific pricing tactics by retailers, such as below-cost selling and volume-related discounts, which raise potential concerns. The Portman Group, however, is unable to offer any regulatory intervention to prevent such tactics or any other aspect of retailers' pricing structures.

Study	Geography	What policy is the AI attempting to influence?	Tactics used	Arguments used
				Furthermore, retailers themselves may find it difficult to self-regulate on pricing because of fears of breaching competition law.... In the off-trade, however, the purchase of extra volume does not necessarily impact on drinking patterns; instead, consumers may choose to store their purchase and consume it at their leisure over weeks or months. For this reason, we are not convinced that action is required to curb volume-related discounts in the off-trade. Any restriction on volume-related discounts in the off-trade risks possible unintended side-effects. Retailers may compete instead by further reducing basic prices or there may be a move to larger-sized stock-keeping units (SKUs); either of these might undermine the intent of the restriction.

Study	Geography	What policy is the AI attempting to influence?	Tactics used	Arguments used
				Educational Initiatives: The Portman Group and its member companies have pioneered a number of inspiring alcohol responsibility initiatives which include: Drinkaware website, labelling, Drinkaware Trust<br><br>No single policy action would transform our drinking culture overnight. Instead, alcohol harm reduction success depends on strong leadership from Government and a properly co-ordinated approach involving the medical profession, teachers, those working in the criminal justice system, regulators, media, parents, retailers and producers. The effect of pricing, advertising and availability are insignificant next to the effect of cultural stereotyping, peer influence and role modelling... We strongly believe that, given

Study	Geography	What policy is the AI attempting to influence?	Tactics used	Arguments used
				accurate and full information, the vast majority of consumers will make healthier lifestyle choices. With others, we need to use more forceful powers of persuasion. In some areas of the UK, the alcohol laws could be enforced to better effect.<br><br>**Scottish and Newcastle**<br><br>We are absolutely committed to working together with the Government, the public health community and industry colleagues to tackle the abuse of alcohol in our society and to promote the responsible consumption of our products.<br><br>S&N UK believes the most effective alcohol strategies should concentrate on tackling alcohol misuse and not consumption per se. Any measures which focus on total consumption obscures differences in how people

Study	Geography	What policy is the AI attempting to influence?	Tactics used	Arguments used
				drink and the outcomes they are likely to experience.<br><br>S&N UK does not believe that new laws or codes are the most effective way to bring about change. There is already a full range of laws in existence to tackle disorder and sales to those who are underage or drunk. We believe strong and consistent enforcement of these laws will be the most effective way forward.<br><br>We strongly believe that industry can be a valuable partner in tackling alcohol misuse, by marketing its products responsibly, providing consumers with information—demonstrated by our full compliance with the voluntary label—and delivering education and responsible drinking programmes.<br><br>However, integral to the

Study	Geography	What policy is the AI attempting to influence?	Tactics used	Arguments used
				success of the strategy is the role of individual responsibility. From Government, to industry to the individual, all parties have a role to play.<br><br>**Wine and Spirits Trade Association**<br><br>It should not be overlooked that there is a significant body of evidence from many global studies that show that moderate alcohol consumption can bring a range of health benefits and therefore tackling alcohol misuse should not be to the detriment of moderate consumers.<br><br>There are difficult problems that link alcohol abuse with social deprivation, unemployment, poor education, poor housing, poor nutrition and other major social issues. As the availability and price of alcohol is the

Study	Geography	What policy is the AI attempting to influence?	Tactics used	Arguments used
				same through the UK, these problems clearly have more complex causes than the freedom with which alcohol can be accessed. It is essential that the underlying causes of these problems be addressed and not solely the regulation of the alcohol industry.<br><br>A targeted approach such is necessary to address the minority that are misusing alcohol without penalising moderate consumers. Whole population approaches to alcohol that concentrate on restricting supply across the board may reduce overall alcohol consumption, but these reductions tend to take place among the wider population rather than the problem drinkers whose consumption puts them at risk of harm.<br><br>Personal responsibility is

Study	Geography	What policy is the AI attempting to influence?	Tactics used	Arguments used
				clearly a key principle when tackling alcohol misuse and one notoriously difficult to foster. We believe that this is best done by greater enforcement of the laws relating to alcohol, quality school education and public health campaigns to promote a responsible attitude to drinking.<br><br>Increasing consumers' knowledge and appreciation of alcoholic products, through tastings and education courses also plays an important role in ensuring people drinks for the right reasons and do so in a healthy way. Wines and spirits can be a fascinating area of interest and teaching consumers to appreciate this is a more effective way of encouraging mature attitudes to alcohol than by demonising it through measures such as separate checkouts and

Study	Geography	What policy is the AI attempting to influence?	Tactics used	Arguments used
				tobacco style health warnings.<br><br>Industry has often shown that its initiatives are able to outpace legislation, as has recently been seen with the implementation of Challenge 21 and Challenge 25 policies, and we believe the Government can secure its policy objective in a more timely and cost-effective way by working with industry rather than against it. Government can support and encourage these voluntary initiatives by avoiding placing additional burdens on industry.<br><br>While industry is involved in a large number of voluntary schemes to reduce the harms caused by alcohol misuse and promote its responsible consumption, it also take seriously the responsibility of marketing its products in a way that does not encourage their

Study	Geography	What policy is the AI attempting to influence?	Tactics used	Arguments used
				misuse. Drinks producers are comprehensively regulated by the BCAP/CAP Advertising Codes (overseen by the Advertising Standards Authority) and the Portman Group's Code of Practice on the Naming, Packaging and Promotion of Alcoholic Drinks. The Portman Group's Code of Practice applies to the naming and packaging of alcoholic drinks and the promotional activities of drinks producers, including press releases, websites and sponsorship. It ensures that such activities are carried out in a socially responsible way. Enforcement of the Independent Complaints Panel's decisions is provided by retailers who do not sell any drink found to be in breach of the Code until that drink's marketing has been altered to comply with the Code. This sanction provides a strong

Study	Geography	What policy is the AI attempting to influence?	Tactics used	Arguments used
				commercial threat to companies, encouraging them to ensure that their marketing is responsible.<br><br>Changing the culture of alcohol consumption in the UK is a complex task that requires the partnership of a range of stakeholders. There is no silver bullet that will solve the problems of alcohol misuse, but by pursuing evidence based policies that target harmful drinkers, addressing problems through better use of the legislation that already exists and by promoting responsible use of alcohol in the population through education, the Government, in partnership with industry and other stakeholders, will be able to make a impact.<br><br>**SABMiller**<br><br>In general terms, moderate

Study	Geography	What policy is the AI attempting to influence?	Tactics used	Arguments used
				drinking is associated with a number of health benefits in some people. Harmful outcomes, on the other hand, are generally associated with heavy drinking patterns and alcohol abuse. But it is important to note that some researchers associate health risks for some people with even moderate levels of consumption.<br><br>The alcohol producer's role includes providing consumers with accurate and balanced reminders about its products, and ensuring that these products are marketed in a way that does not condone or promote irresponsible drinking.... Producers, retailers and government also need to accept that tackling harmful drinking patterns will only be effective if people accept their individual responsibility towards their own alcohol

Study	Geography	What policy is the AI attempting to influence?	Tactics used	Arguments used
				consumption.<br><br>SABMiller supports the Government's voluntary labelling code and was the first alcohol producer to commit to fully implement the Government's voluntary code on labelling... Labelling should be a reminder about levels of unit consumption and key health messages, reinforcing the consumer's existing understanding of responsible alcohol consumption developed from the full provision of accurate, factual information.<br><br>Though we recognise that consumers are ultimately responsible for their own drinking decisions, our advertising will not present refusal, abstinence or moderate consumption in a negative light, suggest that alcohol has curative qualities,

Study	Geography	What policy is the AI attempting to influence?	Tactics used	Arguments used
				depict pregnant women, or be targeted to underage people. In addition to compliance with the legislative codes, SABMiller adheres to the Code on Non Broadcast Advertising, Portman Group Code and the SABMiller Policy on Commercial Communications. When combined with the legislative codes, these voluntary codes help strike the balance between society's expectation for responsible advertising, our right to advertise a legal product in a free and competitive market, and the adult consumer's right to have information about our brands.<br><br>The initial findings of the University of Sheffield's ScHARR Review of the Effects of Alcohol, Price and Promotion raised the concept of "floor prices". These have also been proposed by the

Study	Geography	What policy is the AI attempting to influence?	Tactics used	Arguments used
				Scottish Government in their report Changing Scotland's Relationship with Alcohol. We consider these to be both disproportionate and unnecessary. Minimum prices interfere with the underlying principles of a free market economy. The process of setting minimum prices is arbitrary to which there is no end, and it is predicated on the improbable assumption that raising the price of alcohol will make the minority of drinkers who misuse alcohol and/or engage in anti-social behaviour act differently. Minimum prices are most likely to impact the drinking behaviour of those adults who enjoy drinking alcohol and who do so in a legal, moderate, and socially-acceptable way. In much of continental Europe, the price of alcohol is far cheaper than in the UK but there are not the

Study	Geography	What policy is the AI attempting to influence?	Tactics used	Arguments used
				same problems; societal tolerance (or intolerance) of those who drink irresponsibly or illegally appears to be a greater determinant of the extent to which a country will experience alcohol harm than the price of alcohol.<br><br>A recent study found that the wide availability of social sources, including parents and relatives, meant that reducing availability of alcohol from commercial sources has only a moderate impact on the amount of alcohol consumed by underage drinkers. SABMiller welcomes the British Chief Medical Officer's Guidance on the Consumption of Alcohol by Children and Young People published in January 2009 which advises parents on how to encourage their children, and themselves, to develop a responsible attitude to drinking. This

Study	Geography	What policy is the AI attempting to influence?	Tactics used	Arguments used
				guidance is an important contribution to reducing the harm caused by underage drinking and will hopefully discourage parents, friends and relatives from providing alcohol to those under the legal drinking age.<br><br>Instead of seeking to tackle alcohol misuse through blanket measures such as high prices that unfairly penalise the vast majority of adult drinkers who consume alcohol sensibly and legally, the UK should target the small minority who harm themselves or others when drinking alcohol.<br><br>Tackling harmful drinking patterns will only be effective if people accept their individual responsibility. Increased pricing and restrictions on retailing will not solve the problem of alcohol misuse. These are a small part

Study	Geography	What policy is the AI attempting to influence?	Tactics used	Arguments used
				of a wider issue which has individual judgement and accountability at its heart. While government, public services and industry have a role to play in raising awareness about the potential harm of excessive alcohol consumption, much of the information about alcohol is conveyed through informal channels. In particular, family and peers play a key role in the development of attitudes, awareness and behaviours around drinking. Government and industry initiatives should seek to support them in their role. Parents play a crucial role in teaching their children about the responsible consumption of alcohol. By strengthening their knowledge about alcohol consumption they can ensure that their children grow up to be responsible. Providing parents with accurate and

Study	Geography	What policy is the AI attempting to influence?	Tactics used	Arguments used
				balanced sources of information, such as TalkingAlcohol.com, means they can feel more confident in carrying out this responsibility. Several of SABMiller's businesses offer resources for parents to talk with their children about not drinking, such as Let's Keep Talking in the United States and We Can All Be Parents in Colombia.
Jackson et al, 2000 [15]	UK	General marketing	Other voluntary measures in the United Kingdom are managed by the Portman Group, which was set up in 1990 by the eight major UK drinks manufacturers. Its specific goals are to promote sensible drinking, reduce alcohol-related harm and develop a better understanding of alcohol misuse. It undertakes educational work, much of which is directed at young people. An early initiative of the Portman Group is the launch of its proof-of-age card scheme to help licensees detect under-age drinkers<br><br>Government legislation has been slow to respond to the growth in the youth drinks	

Study	Geography	What policy is the AI attempting to influence?	Tactics used	Arguments used
			sector. The industry has taken the lead, with the Portman Group announcing new industry guidelines for alcoholic soft drinks. The voluntary code recommends the following: a ban on the use of characters or imagery that appeal to under-18s; a ban on containers in anti-social shapes and names that suggest aggression, violence, danger or sexual success; and a requirement that retailers restrict the display of drinks containing alcohol to shelves in alcohol sections only, and do not mix them in refrigerators with soft drinks	
Jernigan, 2011 [16]	Transnational	General marketing	This growing concentration of the beer and distilled spirits industries has created an unprecedented concentration of resources at global and national levels for participating in and influencing policy debates regarding alcohol... To this end, in 1995, 10 of the world's largest distilled spirits and beer marketers at that time (Allied Domecq Spirits and Wine [as of 2006 split up between Pernod Ricard, Diageo, and Beam Global Spirits and Wine], Bacardi-Martini, Brown-Forman, Coors Brewing Company, Guinness PLC [now part of Diageo], Heineken NV, International Distillers and Vintners [now	A CDC systematic review of the literature on the relationship between physical availability of alcohol and health outcomes found "*sufficient evidence of a positive association between outlet density and excessive alcohol consumption and related harms to recommend limiting alcohol outlet density through the use of regulatory authority (e.g., licensing and zoning) as a means of reducing or controlling excessive alcohol*

Study	Geography	What policy is the AI attempting to influence?	Tactics used	Arguments used
			part of Diageo], Miller Brewing Company [now controlled by SAB-Miller, a conglomerate formed by joining Miller with South African Breweries, with the Philip Morris successor company Altria retaining a 20% interest], Joseph E. Seagram & Sons [whose spirits brands were acquired primarily by Pernod Ricard and Diageo in 2000], and South African Breweries [now SABMiller]) banded together to found the ICAP....Miller's involvement in the ICAP as follows:<br><br>*As Miller continues to expand internationally, we will need a better grasp on how different governments may regulate our products ... [this is] the latest initiative in managing worldwide issues, and assisting our sales and marketing group in an increasingly competitive marketplace.*<br><br>In a letter announcing the formation of the ICAP in 1995, Grant outlined 4 goals for the new organization: (1) elaborating a more integrated approach to alcohol policy, involving all interested sectors; (2) developing a common language for promoting more effective dialogue; (3)	*consumption and related harms*". Again, other reviews of the global literature have corroborated this finding. The ICAP review of the same literature states that ''a debate has been developing around the effectiveness of availability control measures,'' claims that ''[t]here is evidence that efforts by those desiring to circumvent existing controls has fueled organized crime'' (with the cited source being an article by a Diageo employee published in another ICAP collection)<br><br>A subsequent ICAP brochure described the first goal as an effort to reassess ''current theories with a primary focus on the differences between positive and negative patterns of drinking.'' This emphasis on the patterns of drinking (as opposed to population levels of consumption) and positive

Study	Geography	What policy is the AI attempting to influence?	Tactics used	Arguments used
			encouraging initiatives designed to meet the needs of developing countries; and (4) promoting responsible lifestyles – A subsequent ICAP brochure described the first goal as an effort to reassess ‘‘current theories with a primary focus on the differences between positive and negative patterns of drinking.’’ This emphasis on the patterns of drinking (as opposed to population levels of consumption) and positive effects of alcohol use would be a major ICAP focus in its first decade, developed in a 1998 conference titled ‘‘Permission for Pleasure,’’ and a subsequent edited collection of essays titled, Alcohol and Pleasure: A Health Perspective. ‘‘Involving all interested sectors’’ would in practice mean pushing for and engaging in active alcohol industry involvement in public health policymaking regarding alcohol, directing debate over alcohol policy into areas where the alcohol industry could agree, and thus focusing on education and identification and treatment of the heaviest drinkers (among the least effective and least cost-effective approaches to alcohol problems) and staying away from population-level strategies such	effects of alcohol use would be a major ICAP focus in its first decade, developed in a 1998 conference titled ‘‘Permission for Pleasure,’’ and a subsequent edited collection of essays titled, Alcohol and Pleasure: A Health Perspective. ‘‘Involving all interested sectors’’ would in practice mean pushing for and engaging in active alcohol industry involvement in public health policymaking regarding alcohol, directing debate over alcohol policy into areas where the alcohol industry could agree, and thus focusing on education and identification and treatment of the heaviest drinkers (among the least effective and least cost-effective approaches to alcohol problems) and staying away from population-level strategies such as increased taxes or restrictions on

Study	Geography	What policy is the AI attempting to influence?	Tactics used	Arguments used
			as increased taxes or restrictions on marketing or physical availability. The second goal would seek to remove phrases troubling to the industry such as ‘‘alcohol and other drugs’’ from the official lexicon...The third would aim to protect the industry’s ability to expand in areas where its potential for growth was greatest, by influencing and encouraging weak alcohol policies in this region. The fourth goal would in practice mean promoting drinking and the drinker’s right to obtain alcohol.<br><br>Much of the ICAP’s activities have focused on countering the influence of the WHO and leading alcohol researchers by essentially functioning like a WHO unit on alcohol, with certain key omissions. Building on Grant’s decade of experience at the WHO in creating and distributing edited collections of contributions by scholars from around the world, the ICAP would commission and produce 10 such book-length collections between 1998 and 2010, as well as 2 other monographs, 6 briefin papers for consultation with the WHO, 20 brief issue reports, 4 in-depth ICAP reviews of issues in alcohol policy, 5 periodic reviews of drinking	marketing or physical availability.<br><br>The ICAP report on drinking age limits states that some ‘‘argue that a minimum drinking age of 21 is impractical’’ and that ‘‘the emphasis should be less on stigmatizing alcohol and more on promoting responsible consumption of alcohol.’’

Study	Geography	What policy is the AI attempting to influence?	Tactics used	Arguments used
			and culture, 8 peer-reviewed journal articles written by ICAP staff and paid consultants, 1 special issue of a journal devoted to alcohol and harm reduction, and 22 charters, working papers, progress reports, and other brief policy statements or guides to policy implementation. It also produced 4 policy guides, 9 health briefing papers, 8 issue briefing papers, and 4 policy tool kits, ''guides for implementation of interventions to reduce harmful drinking.' (and Table 3)<br><br>Although the WHO was producing fewer publications during this period, several ICAP publications seemed to attempt to counter or pre-empt similar WHO publications. For instance, in 1994 the European office of the WHO had sponsored a group of 17 scientists from 9 countries to produce a comprehensive review of the global research literature on alcohol and public health. The book made a strong, evidence-based argument for population-level strategies such as excise tax increases and controls over physical availability. The ICAP's first policy manifesto appeared in 1998, and was titled *Drinking Patterns and Their Consequences*. It sought to reframe the debate from societal	

Study	Geography	What policy is the AI attempting to influence?	Tactics used	Arguments used
			measures to individual patterns of drinking, which could be harmful or beneficial. This reframing also shifted the focus from the product and the practices of the industry to the behavior of individual drinkers..... Whereas WHO estimates have placed alcohol's role in the global burden of disease on a par with that of tobacco, the ICAP, in a publication of its own titled Alcohol Consumption and the Burden of Disease, focused on the limitations of the study, including claiming (incorrectly) that the estimates had failed to take into account different patterns of drinking....Two other ICAP publications directly addressed the well-documented public health strategies of increasing alcohol taxes and restricting physical availability.<br><br>The ICAP has also produced a series of briefing papers, reviews of alcohol policy issues that claim to be surveys of the research literature... The ICAP papers focus on the disagreements and inconclusiveness of alcohol policy research. For instance, the ICAP briefing paper on health warning labels on alcohol reflects ''the equivocal nature of the contemporary HWL [health warning	

Study	Geography	What policy is the AI attempting to influence?	Tactics used	Arguments used
			label] debate.'' .... Other reports reflect the alcohol industry's interest in promoting alcohol consumption. For instance, the ICAP report on safe drinking levels concludes by noting that ''both the UK and the US guidelines draw attention to the health benefits of moderate alcohol consumption.''<br><br>The ICAP developed its own ''blue book,'' an Internet-based set of ''practical guides for alcohol policy and targeted interventions.'' ...Described as ''a new way to address the role of alcohol in society,'' the blue book offers 23 ''modules'' for policy development. Conspicuously missing from these modules is any mention of 3 of the most effective policy approaches to alcohol problems: taxation, restrictions on advertising and marketing, and limits on physical availability.<br><br>Finally, the industry must consistently emphasize alcohol education. As Miller's vice president of corporate relations told a meeting of alcohol industry executives in 1996, when the beer company was still under the control of Philip Morris, *"First, we must continue to educate consumers to drink our products responsibly ... . Second, we must*	

Study	Geography	What policy is the AI attempting to influence?	Tactics used	Arguments used
			continue to educate the public ... that there is a vast difference ... between consumption ... and abuse ... of our products ... and between alcohol ... and illegal drugs ... . And third, we must continue to educate policy makers ... that we ... and the 100 million Americans who drink alcohol beverages ... don't need higher taxes ... and more restrictive regulations ... . For our industry, a positive image ... based on accurate information about our products ... is not a luxury ... but a necessity ... a necessity for survival ... . This is hardball ... and we've got to play to win.''	
Monardi and Glantz, 1998 [17]	USA (Wisconsin)	In July, 1997 ... proposal that most tobacco and alcohol advertisements on billboards be ended in the City of Milwaukee.		Mike Brophy, communications director for Miller Beer, expressed concern for reducing access to cigarettes and alcohol to children, but found it odd that the alderman would introduce a proposal that would "demonize" a product (beer) that was a significant part of Milwaukee's history.
Mosher, 2012 [18]	USA	General marketing	Diageo initiated a sophisticated public relations program during the same period that it launched the Smirnoff brand	The code, established in1997, is periodically updated and states that the company's

Study	Geography	What policy is the AI attempting to influence?	Tactics used	Arguments used
			campaign. Its purpose was to convince policymakers, public health and medical groups, and the public that the company was committed to deterring underage drinking and other social harms associate with its products. Diageo hired Guy Smith, a former vice president of Philip Morris, a veteran of the tobacco wars, and a former advisor to President Clinton, to head up its marketing public relations division, which would design and implement the campaign. The following strategies were used.<br><br>1. Establish a self-regulatory structure to monitor the company's alcohol advertising. Diageo established its own responsible marketing code, which it describes as ''a beacon for responsible marketing and brand innovation'' according to DrinkIQ. The code, established in1997, is periodically updated and states that the company's advertising must<br><br>*Be aimed only at adults and never target those younger than the legal purchase age for alcohol [and] be designed and placed for an adult audience, and never be designed or constructed or placed in a way that appeal*	advertising must<br><br>*Be aimed only at adults and never target those younger than the legal purchase age for alcohol [and] be designed and placed for an adult audience, and never be designed or constructed or placed in a way that appeal primarily to individuals younger than the legal purchase age for alcohol.*

Study	Geography	What policy is the AI attempting to influence?	Tactics used	Arguments used
			primarily to individuals younger than the legal purchase age for alcohol.<br><br>2. Broadcast ''responsibility'' advertisements. Between 2001 and 2005, Diageo spent 17.7% of its advertising budget on responsibility television advertising, which is focused primarily on educating viewers about how to prevent underage drinking and drunk driving– –far more than any other alcoholic beverage producer. Even so, underage youths were far more likely to see a Diageo product advertisement than a responsibility advertisement during this period.<br><br>3. Fund prevention programs that focus on education, public awareness, and responsible retail practices. According to Diageo's 2009 Corporate Citizenship Report,<br><br>*We support practical programmes in many of our markets to tackle particular examples of harm from alcohol misuse... .This year we led or supported over 130 such initiatives in over 40 countries. The Diageo Responsible Drinking Fund, which had resources this year of £400,000, provided financial support,*	

Study	Geography	What policy is the AI attempting to influence?	Tactics used	Arguments used
			backed up by expert guidance.<br><br>Examples include DrinkIQ, a Web site providing information on alcohol and alcohol problems, programs focused on responsible alcohol retail practices, and drinking and driving and underage drinking awareness programs.<br><br>4. Build partnerships with medical and public health organizations and government agencies. According to Diageo, most of its funded programs are<br><br>*undertaken in partnership with other organizations including governments, non-governmental organizations, universities, researchers, physicians, [and] law enforcement.*<br><br>Medical and public health groups are a high priority for building partnerships; governmental agencies are equally important. For example, Diageo has reached out to the Federal Trade Commission, which regulates alcohol advertising and called for the industry to support the Federal Trade Commission's ''We Don't Serve Teens	

Study	Geography	What policy is the AI attempting to influence?	Tactics used	Arguments used
			Campaign.'' The company's cooperation with governmental agencies on prevention efforts dovetail with Diageo's extensive political lobbying activities. Diageo and other distillers have also sponsored scientific research and sought to influence public perceptions regarding research findings.<br><br>5. Establish industry-based ''social aspects'' organizations. Over the past decade, Diageo has helped organize and fund 14 social aspects organizations worldwide, including the Century Council and the International Center on Alcohol Policy in the United States. These industry-member organizations sponsor programs similar to those funded by the Diageo Responsible Drinking Fund and further the goals of the Diageo public relations campaign. Social aspects organizations serve the long-term marketing interests of their industry members.<br><br>These components fit a strategy used by several industries that have products with potential public health harms: they promote ineffective self-regulatory programs, discourage governmental regulation, broadcast a message of corporate	

Study	Geography	What policy is the AI attempting to influence?	Tactics used	Arguments used
			responsibility, fund programs with public relations value that do not interfere with marketing, recruit potential opponents with corporate funds, and seek opportunities to cooperate with governmental agencies that might otherwise interfere with marketing goals.	
Munro and De Wever, 2008 [19]	Australia	2003 - Ministerial Council on Drug Strategy (MCDS) reviewed the effectiveness of ABAC (Alcohol Beverages Advertising Code)	2003 review: The ad hoc National Committee for the Review of Alcohol Advertising (NCRAA) concluded that ABAC 'does not address public health concerns about alcohol advertising and use' ..... NCRAA determined that the ASB did not have sufficient understanding of public health concerns about alcohol advertising to make adequate judgements about specific advertisements<br><br>Rather than impose a different system, MCDS allowed the industry to continue to conduct an ABAC system that was 'revised' and 'strengthened' by 25 amendments	Alcohol advertising in Australia has been self-regulated by peak industry bodies through the ABAC since 1998 [National Health Policy on Alcohol warned that a mandatory form of regulation was an option if self-regulation failed] .... The decision in 2003 by the Ministerial Council on Drug Strategy (MCDS) to review the effectiveness of ABAC indicated it had lost confidence in the code, and the subsequent investigation found the ABAC system was deficient, even dysfunctional. Yet throughout 5 years of operation the ABAC principals had maintained the system

Study	Geography	What policy is the AI attempting to influence?	Tactics used	Arguments used
				represented best practice and could not be improved. The Distilled Spirits Industry Council of Australia (DSICA) had proclaimed: '. . .Australia has a comprehensive self regulatory system in place that specifically prevents advertising directed at young people'; '. . . the Pre-vetting system has been extraordinarily successful' and it had '. . . proved ground breaking internationally with several countries adopting the model'. The rigour of the system was lauded: 'the industry's voluntary code and its complaints system was one of the world's most stringent'; and 'Australian brewers . . . lead the world in strong self regulation of advertising'
Nelson, 2010 [20]	OECD	General marketing		The standard response from the alcohol industry has been that advertising affects brand and beverage shares, and

Study	Geography	What policy is the AI attempting to influence?	Tactics used	Arguments used
				content issues can be dealt with through industry self-regulation and voluntary content-placement codes
Parry, 2010 [21]	South Africa	Ministry gave the go-ahead for taking up the issue of restrictions on alcohol advertising in 2002–04	They joined forces with the Freedom of Commercial Speech Trust ... This Trust was established in 1997 soon after the health minister had announced her decision to regulate tobacco marketing.	The liquor industry argued that self-regulation was sufficient
Thamarangsi, 2008 [22]	Thailand	General marketing	In 2006, alcohol industry representatives, together with the entertainment venues surrounding a University, declared a code of practice not to sell alcohol to students in uniform during the daytime. This was intended to demonstrate self-regulation as an alternative to the total advertising ban which was being considered at the time.<br><br>Many stakeholders can alter the implementation outcomes by acting as the co-implementers. These include the role of the alcohol industry and related sectors in marketing self-regulation, the alcohol industry as a partner of the DDD Foundations, and the media in public	A decrease in alcohol-generated benefits, of the negative impacts of alcohol policy particularly in economic terms, is a common policy discourse for the industry. For example, the CEO of an alcohol company claimed that a total advertising ban would create 100,000 unemployed: “These measures do not attack the root cause of the problem. They will destroy alcohol industry by the misconception that all beverages are bad and are the cause of all problems...

Study	Geography	What policy is the AI attempting to influence?	Tactics used	Arguments used
			campaigns. However, many see that these approaches may be designed to divert policy direction and to promote the corporate image.<br><br>In many instances, alcohol entrepreneurs negotiated ‘trade-offs’ with policy makers, such as ‘using sport as a hostage’. They threatened to withdraw sports sponsorship in retaliation for the advertising ban.<br><br>The Federation on Alcohol Control of Thailand (FACT) was established during the formulation process for the FDA advertising ban regulation, in October 2006. Therefore, it was clearly seen as a lobbyist from the beginning. Its members include alcohol producers, sellers and distributors, the Thai Retail Association, The Marketing Association of Thailand, the Thai Hotel Association, the Thai Restaurant Association, and the Tourism Association..... FACT opposed the regulation in an aggressive fashion, including warning on the impacts to employment, criticising it as rushed, fishy, and unfair. They also threatened to petition the Administrative court.	The advertising sector will lose 1.5 to 2 billion Baht (by the ACC Bill)... Related sectors such as bottle shops, restaurants and tourism will be affected by at least 10% of about ten billion Baht” (Suthabodi Sattabut – Assistant Manager Boonrawd Brewery)<br><br>The alcohol industry used this position (advertising ban is counter to the concept of trade competition, individual freedom and trade freedom) to oppose the ACC Bill.<br><br>A group of alcohol importers protested to the Thai Government, claiming that the advertising regulations were contrary to the WTO agreement.<br><br>The alcohol industry have used the example of increasing overall consumption in some countries that have banned

Study	Geography	What policy is the AI attempting to influence?	Tactics used	Arguments used
			Foreign alcohol entrepreneurs have had a more explicit role in the alcohol policy process including lobbying for their participation. They were key particiapants in the meeting of 30 entrepreneurs in July 2003 to oppose advertising regulation and were criticised for delaying the decision.<br><br>In mid 2006, the US Ambassador took tobacco and alcohol entrepreneurs to meet with the Health Minister. In late 2006, the French Ambassador together with a few alcohol companies submitted their request to delay the regulation of advertising: "The alcohol companies, including whiskey giant Johnnie Walker, said they wanted to have a say in the public hearings on the new Bill being drafter to completely ban all forms of alcohol advertising" [Minister of PH after meeting with US Ambassador and alcohol industry representatives, 2006].<br><br>In many cases, alcohol operators used windows of opportunity in the alcohol policy process to undermine their business opponents, for example by deviating policy direction during the advertising ban formulation to advocate for White spirits tax	alcohol advertising to oppose such interventions.<br><br>"We advocate for a self regulatory system among alcohol producers as we know that advertising bans are ineffective from experience of New Zealand, US, Sweden and Denmark" (an administrator of an international alcohol company, 2005)<br><br>The alcohol industry claimed the partial advertising regulation was a failure and later called for its repeal.<br><br>...alcohol entrepreneurs used the failure to reduce traffic accidents in the 2004 New Year to call for the repeal of the partial advertising ban<br><br>Most policy players seem to agree about the situation of poor policy enforcement. However the alcohol industry's

Study	Geography	What policy is the AI attempting to influence?	Tactics used	Arguments used
			rate increases.<br><br>Following the collective movement against the partial advertising ban, six foreign alcohol operators established REACT (Responsible & Ethical Alcohol Consumption in Thailand) in 2004... one particularly innovative approach from REACT was to use technical mechanisms such as organising technical seminars, announcing a study of the impact of alcohol advertising and the effectiveness of the advertising ban...	agenda in calling for stronger enforcement is possibly to avoid the enactment of stronger alcohol legislation. For example, industry representatives recommended that the government strengthen the enforcement of the existing laws in a lobby against the 2003 partial advertising ban.<br><br>Foreign entrepreneurs ...labelling the Thai government a dictatorship because of the advertising ban<br><br>A Riche Monde administrator claimed that REACT's self-regulation has proved its effectiveness, and it a better alternative to the total ban [on sports advertising]<br><br>FACT opposed the regulation in an aggressive fashion, including warning on the impacts to employment,

Study	Geography	What policy is the AI attempting to influence?	Tactics used	Arguments used
				criticising it as rushed, fishy, and unfair.
van Hoof et al, 2008 [23]	Netherlands	General marketing	In the current Dutch situation, happy hours and other alcohol discounts in the catering industry are subject only to self-regulation. The Dutch Foundation for the Responsible Use of Alcohol (STIVA), an organization of alcohol industries representatives, has formulated an Advertising Code containing rules for alcohol promotions. According to the Advertising Code, alcohol promotions are not allowed in premises where 25% or more of the visitors are minors. Nor are premises permitted to offer alcoholic beverages for free or to sell them for less than half of the normal price, to offer more than one discounted drink per customer, or to combine alcohol discounts with additional free gifts.	

Explanation of systematic searches

Search string: (corporat* OR industr* OR compan* OR busines* OR firm*) AND (alcohol OR drink) AND (marketing OR advertis* OR sponsor*) AND (regulat* OR policy OR legislat*)

Table 5: Searches completed and the number of articles returned

Location of search	Types of publications available	Search notes	Articles returned *(excluding duplicates)*
Web of Knowledge (which included Web of Science, BIOSIS Previews and MEDLINE)	Journal articles, conference proceedings, websites, reports, books, letters, and meeting transcripts	Full string in 'Topic'	**418**
Business Source Premier	Journal articles, magazines, trade publications, market research reports, industry reports, country reports, company profiles, and conference proceedings	Full string in 'Topic', except '(alcohol OR drink)' in 'Title' (searching with it in 'topic' returned far too many hits)	**297**
EMBASE	Journal and peer-reviewed articles, and conference abstracts	Full string in 'Topic'	**144**
Contacting experts	All	E-mail	**16**
Search engine 'Google'	All	Full string	**42**
		**Total**	**917**

References

1. LOHMANN, S., 1998. *An information rationale for the power of special interests.* American Political Science Review: p. 809-827.
2. HILLMAN, A.J. and HITT, M.A., 1999. *Corporate political strategy formulation: A model of approach, participation, and strategy decisions.* Academy of Management Review: p. 825-842.
3. LORD, M.D., 2003. *Constituency Building as the Foundation for Corporate Political Strategy.* The Academy of Management Executive (1993-2005). 17(1): p. 112-124.
4. SAVELL, E., GILMORE, A.B., and FOOKS, G., 2014. *How Does the Tobacco Industry Attempt to Influence Marketing Regulations? A Systematic Review.* PLOS ONE. 9(2).
5. CROSBIE, E.M.A., SEBRIE, E.M.M.D.M.P.H., and GLANTZ, S.A.P., 2012. *Tobacco Control Legislation in Costa Rica (1971-2012): After 40 Years of Tobacco Industry Dominance, Tobacco Control Advocacy Succeeds*.
6. FOOKS, G. and GILMORE, A., 2013. *International trade law, plain packaging and tobacco industry political activity: the Trans-Pacific Partnership.* Tobacco Control. online first.
7. B&T MAGAZINE, 2012. *Does There Need to be Greater Regulation of Alcohol Advertising?*, in *B&T Magazine*.
8. BAKKE, O. and ENDAL, D., 2010. *Vested Interests in Addiction Research and Policy - Alcohol policies out of context: drinks industry supplanting government role in alcohol policies in sub-Saharan Africa.* Addiction. 105(1): p. 22-28.
9. BOND, L., DAUBE, M., and CHIKRITZHA, T., 2009. *Access to confidential alcohol industry documents: from 'Big Tobacco' to 'Big Booze'.* Australasian Medical Journal. 2(3): p. 1-26.
10. CASSWELL, S. and THAMARANGSI, T., 2009. *Reducing harm from alcohol: call to action.* Lancet. 373(9682): p. 2247-2257.
11. FOGARTY, A.S. and CHAPMAN, S., 2012. *Advocates, interest groups and Australian news coverage of alcohol advertising restrictions: content and framing analysis.* Bmc Public Health. 12(1): p. 727.
12. GIESBRECHT, N., 2000. *Roles of commercial interests in alcohol policies: recent developments in North America.* Addiction. 95(12): p. S581-S595.
13. HOPE, A., 2006. *The influence of the alcohol industry on alcohol policy in Ireland.* NORDISK ALKOHOL OCH NARKOTIKATIDSKRIFT. 23(6): p. 467.
14. HOUSE OF COMMONS: HEALTH COMMITTEE, 2010. *Alcohol: First Report of Session 2009-10, Volumes 1, 2 and Written Evidence*. The Stationary Office: London.
15. JACKSON, M.C., et al., 2000. *Marketing alcohol to young people: Implications for industry regulation and reserach policy.* Addiction. 95 (SUPPL. 4): p. S597-S608.
16. JERNIGAN, D.H., 2012. *Global alcohol producers, science, and policy: the case of the international center for alcohol policies.* Journal Information. 102(1).
17. MONARDI, F.M. and GLANTZ, S.A., 1998. *Tobacco Industry Political Activity and Tobacco Control Policy Making in Wisconsin: 1983-1998*.
18. MOSHER, J.F., 2012. *Joe Camel in a bottle: Diageo, the Smirnoff brand, and the transformation of the youth alcohol market.* American Journal of Public Health. 102(1).
19. MUNRO, G. and DE WEVER, J., 2008. *Culture clash: Alcohol marketing and public health aspirations.* Drug and Alcohol Review. 27 (2): p. 204-211.
20. NELSON, J.P., 2010. *Alcohol advertising bans, consumption and control policies in seventeen OECD countries, 1975-2000.* Applied Economics. 42(7): p. 803-823.

21. PARRY, C.D.H., 2010. *Alcohol policy in South Africa: a review of policy development processes between 1994 and 2009.* Addiction. 105(8): p. 1340-1345.
22. THAMARANGSI, T., 2008. *Alcohol Policy Process in Thailand*. Massey Univerisity, Auckland, New Zealand.
23. VAN HOOF, J., VAN NOORDENBURG, M., and DE JONG, M., 2008. *Happy hours and other alcohol discounts in cafes: Prevalence and effects on underage adolescents.* Journal of Public Health Policy. 29(3): p. 340-352.
